# Mechanistic Insights Into Nitric Oxide Capture and Release in a Radical‐Scavenging Zinc Ascorbate Metal–Organic Framework

**DOI:** 10.1002/smsc.70312

**Published:** 2026-05-18

**Authors:** Tia Kristian Tajnšek, Petar Djinović, Matej Huš, Blaž Likozar, Mariana Pimenta Lopes, Sílvia Carvalho, Mary Batista, Moises Luzia Pinto, João Pires, Safiyye Kavak, Sara Bals, Simon Caserman, Nataša Zabukovec Logar, Matjaž Mazaj

**Affiliations:** ^1^ National Institute of Chemistry Ljubljana Slovenia; ^2^ University of Nova Gorica Nova Gorica Slovenia; ^3^ Association for the Technical Culture of Slovenia (ZOTKS) Ljubljana Slovenia; ^4^ Institute of the Protection of Cultural Heritage of Slovenia (ZVKDS) Ljubljana Slovenia; ^5^ CERENA Department of Chemical Engineering Instituto Superior Técnico University of Lisbon Lisbon Portugal; ^6^ CQE – Centro de Química Estrutural Institute of Molecular Sciences Departamento de Química e Bioquímica Faculdade de Ciências University of Lisbon Lisbon Portugal; ^7^ EMAT and NanoLight Centre of Excellence Faculty of Science Department of Physics University of Antwerpen Antwerp Belgium

**Keywords:** gas adsorption mechanism, metal–organic frameworks, nitric oxide storage and release, radical scavenger, zinc ascorbate

## Abstract

Efficient capture and controlled release of nitric oxide (NO) are essential for therapeutic gas delivery. Here, we investigate NO sorption in bioNICS‐1, a zinc‐based ascorbate metal–organic framework (MOF), and its propionic acid‐modified analogue (bioNICS1‐actPA). Structural modulation enhances NO uptake and retention, as evidenced by equilibrium isotherms and pronounced desorption hysteresis that reflect strong host–guest interactions. PXRD and TEM studies reveal adaptive unit‐cell distortions upon adsorption, while kinetic and density functional theory (DFT) calculations identify multiple binding sites with distinct interaction strengths. Controlled‐release experiments demonstrate humidity‐triggered desorption, enabling finely regulated NO delivery. This work uncovers a previously unreported NO binding mechanism in MOFs and establishes radical‐scavenging linkers as a powerful design concept for targeted gas capture, positioning bioNICS‐1 as a versatile platform for next‐generation therapeutic gas delivery technologies.

## Graphical abstract

A zinc–ascorbate MOF (bioNICS‐1) enables tuneable nitric oxide (NO) capture and humidity‐triggered release through defect engineering and structural modulation. Organic acid treatment enhances binding site accessibility and retention strength. BioNICS‐1 offers controlled NO delivery, positioning it as a promising platform for therapeutic gasotransmitter applications in biomedical environments.

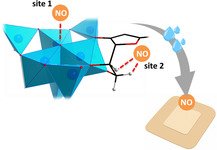

## 1 Introduction

Metal–organic frameworks (MOFs) composed of metal units coordinated with organic ligands possesses highly tuneable structures with large surface areas and pore volumes suitable for gas storage and controlled release [1]. In recent years, MOFs have emerged as promising platforms for the delivery of therapeutic gases, particularly nitric oxide (NO)—a key signalling molecule involved in vascular regulation, neurotransmissions, angiogenesis, inflammation, and wound healing [2]. Through rational design and functionalization, MOFs can be rendered biocompatible and tailored to encapsulate and release NO with precise control over dosage and kinetics [3, 4, 5]. Among accessible release mechanisms, water‐triggered displacement is the most relevant for biomedical applications, as it allows physiological activation without external stimuli such as heat or UV light [1]. There is another step in the gas adsorption cycle: reactivation. However, unlike classic gas adsorption for air purification [6] or CO_2_ capture [7], therapeutic gas adsorbents do not necessarily need to be, nor are they expected to be, recyclable. Selectiveness in gas adsorption is also not a priority in the biomedical case, since no other gas (except therapeutic gas) would be present during loading (adsorption).

Most extensively studied MOFs for NO loading and delivery are the materials containing coordinatively unsaturated metal sites (CUSs) [3, 4, 8, 9, 10, 11]. These CUSs may be intrinsic to the material [4, 12] or generated through defect engineering [13], enabling direct NO coordination. Post‐synthetic functionalization of CUSs with secondary amines can yield NONOate‐type donors upon NO exposure [14], while metal‐nitrite complexes, as reported for MIP‐177(Ti), can also be formed [5]. In contrast, linker‐based NO adsorption and delivery strategies have received comparatively less attention. Examples include N‐nitrosoamine‐functionalised linkers (e.g. NOF‐11 and NOF‐12) [15], diazeniumdiolates forming linkers [16], and amino‐functionalised MOFs such as UiO‐66‐NH_2_ [17, 18] or HKUST‐1 derivatives [19], where ligand chemistry strongly influences both adsorption and release profiles. Notably, in HKUST‐1, linker modification yields cooperative effects between CUSs and linker functionalities, enhancing NO binding.

There is, however, another avenue that is still highly unexplored regarding NO loading and delivery with MOFs – the use of radical scavengers (RS) as functional linkers. RS neutralise or quench reactive oxygen (ROS) or reactive nitrogen species (RNS) in biological environments by donating electrons or hydrogen atoms to stabilise radicals. Although many compounds can act as scavengers, they must meet additional criteria in order to function in an organism [20]. RS are crudely divided into endogenous (enzymatic and non‐enzymatic) and exogenous scavengers – representative examples are listed in Table S1. Most of them are considered to be broad‐spectrum, reacting with diverse ROS/RNS species [21, 22]. Importantly, many of these RS possess coordination‐capable functional groups, making them viable as MOF linkers.

As linkers are spatially separated within a MOF lattice, there is a high possibility that RS would retain their intrinsic‐scavenging activity and quench free radicals whilst being a part of the MOF structure.

While research on enzyme – MOF combination is focused on, either the delivery of enzymes with MOFs [23] or MOFs mimicking the natural enzyme function [24], some other MOFs do utilise exogenous RS as linkers for other purposes. For instance, metalloporphyrinic MOFs used in photodynamic cancer therapy rely on ROS/RNS generation rather than scavenging [25]. Zr‐based PCN‐223 MOF incorporating L‐arginine (an NO‐precursor) has been explored for catalytic NO formation [26], while Mg‐gallate MOFs have shown promise in microneedle patches for diabetic wound healing [27]. Although gallic acid retains its radical‐scavenging function upon release, NO loading was not investigated. Ellagic acid has been used to construct several MOFs [28], but their therapeutic gas potential remains unexplored. Medi‐MOF‐1 [29] (Zn‐curcumin) exhibits interesting gas sorption behaviour, but has not been evaluated for NO delivery [30].

Recently, our group reported bioNICS‐1, the only known bioMOF constructed from ascorbic acid – one of the most potent natural RS – coordinated to Zn(II) [31]. This framework combines permanent microporosity, a suitable toxicological profile, and potential NO‐binding sites with both the ligand and CUSs, making it a strong candidate for NO capture and controlled release [32, 33].

Herein, we investigate the NO binding and release behaviour of bioNICS‐1 and its defect‐engineered analogue, focusing on how structural modification via carboxylic acid treatment influences adsorption kinetics, framework interactions, and water‐triggered NO release. Through a combination of experimental and computational studies, we established how the integration of redox‐active ascorbic acid linkers within a crystalline Zn(II) framework enables a dual‐function design for tuneable NO storage and delivery, providing a versatile platform for future biomedical gasotransmitter applications.

## 2 Results and Discussion

The study was motivated by the hypothesis that the redox‐active moiety of the radical‐scavenging linker remains accessible and chemically functional while coordinated to the metal nodes. If preserved, this functionality could contribute directly to NO capture, complementing adsorption at the CUSs through combined linker–NO and CUS–NO interactions.

**FIGURE 1:**
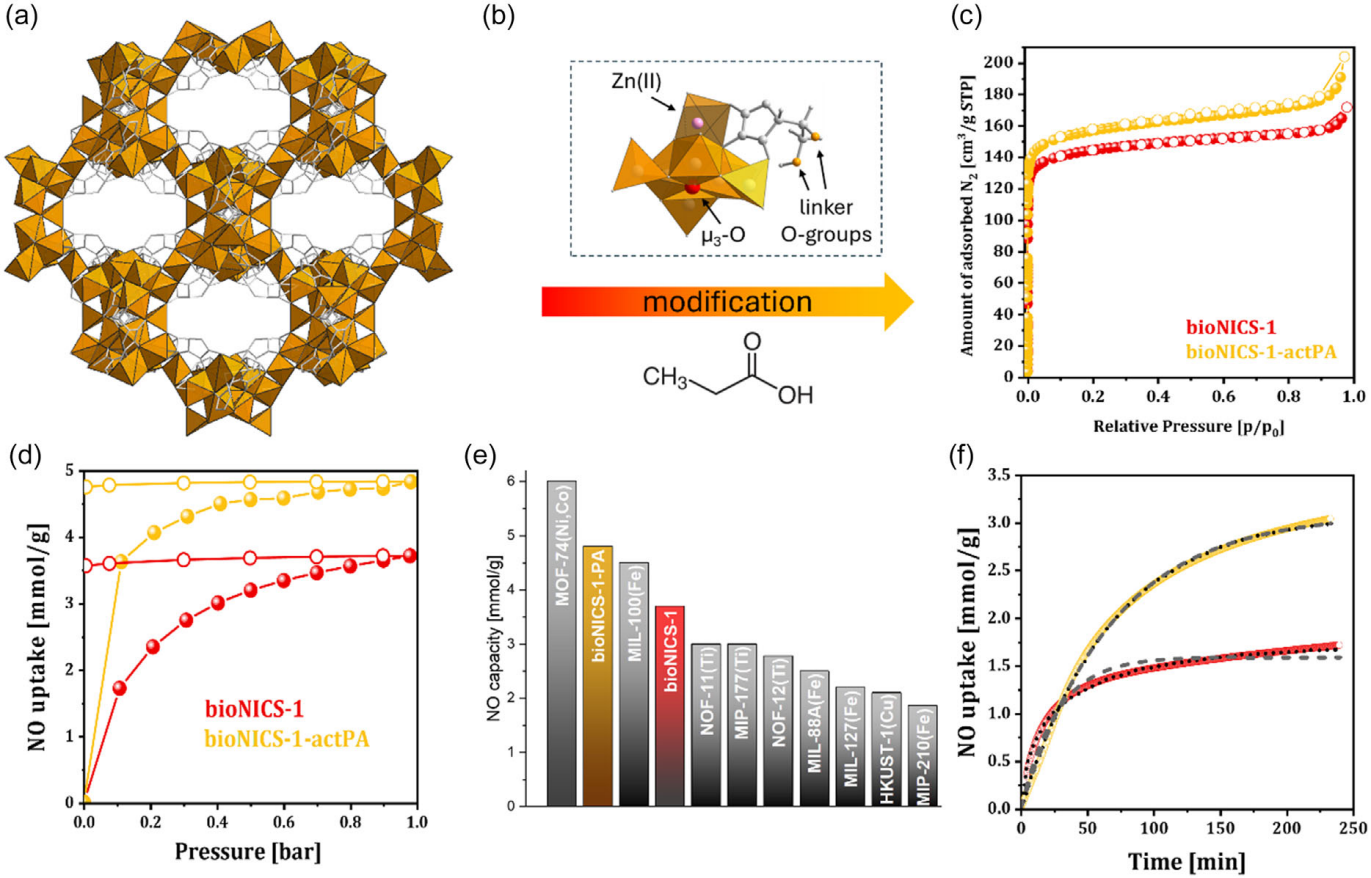
(a) Crystal structure view of bioNICS‐1 along the [100] microporous channels showing the three‐dimensional Zn‐ascorbate framework constructed from interconnected Zn‐O polyhedra and bridged through ascorbate ligands; (b) local coordination environment around Zn(II) nodes with indicated potential NO adsorption sites; (c) effect of propionic acid (PA) modulation on porosity, evidenced by increased BET surface area derived from N_2_ adsorption isotherms at 77 K which can also enhance accessibility of NO adsorption sites; (d) NO adsorption–desorption isotherms measured at 25°C up to 1 bar, showing improved NO capacity upon PA modulation (adsorption: full symbols, desorption: empty symbols); (e) comparison of NO uptake capacities for bioNICS‐1 materials with representative MOFs reported under ambient conditions, highlighting competitive performance of bioNICS‐1 with the state‐of‐the‐art materials; and (f) NO adsorption kinetics upon dosing to 100 mbar at 25°C (experimental data: circles; Avrami model fit: dotted lines; linear driving‐force model: dashed lines), showing distinct adsorption regimes for pristine and modulated frameworks.

The Zn–ascorbate MOF bioNICS‐1 was selected as the model system due to its distinctive structural and functional properties. Its three‐dimensional Zn‐based inorganic building unit is bridged by the ascorbic acid linker, forming a permanently microporous framework with accessible acid binding sites [31] ‐ Figure 1a, b. Additionally, a recent study showed that the ascorbate linker retains its intrinsic antioxidant activity even within the bioNICS1 framework [34], supporting the premise that its radical‐scavenging capability remains preserved.

To further optimise bioNICS‐1 for NO uptake and controlled release, defect engineering was employed as a structural tuning strategy. Previous work demonstrated that porosity and acid site density can be modulated by introducing point defects through the addition of propionic acid (PA), a well‐established coordination modulator [32, 35], during the synthesis [36]. In the present study, we confirmed that comparable modifications can also be induced post‐synthetically via mild acid treatment (Table S2 and Figures S1–S6). Although the PXRD patterns of PA‐modified samples closely resemble that of the pristine bioNICS‐1 (Figure S1), indicating maintained long‐range order, conventional diffraction cannot detect local disorder. Modulator‐induced defects – such as missing linkers or node vacancies – can significantly alter the local chemical environment, generating additional open metal sites or acid functionalities that critically affect host–guest interactions.

PA modification increased the BET surface area from 573 m^2^/g for pristine bioNICS‐1 to 620 m^2^/g for bioNICS‐1‐actPA (Figure 1c and S4) and may also introduced additional microporosity (Figure S5 and S6). Interestingly, the treatment did not markedly change the overall acidity of the material, as indicated by NH_3_ adsorption isotherms (Figures S7 and S8). However, a small increase in the chemisorbed ammonia fraction in the PA‐modified sample (Figure S8) suggests the presence of new or more accessible acid sites. Adsorption of ammonia did not cause any measurable structural changes, as verified by PXRD (Figure S9).

### 2.1 NO Adsorption Process and Kinetics

Equilibrium isotherms for NO adsorption were measured at 25°C up to 1 bar (Figure 1d and S10) to evaluate the adsorption capacity and behaviour of pristine and PA‐modified bioNICS‐1 materials. The unmodified bioNICS‐1 exhibits a Langmuir‐type isotherm, characterised by a gradual increase in adsorption, approaching saturation at approximately 3.7 mmol/g. The modified analogue, bioNICS‐1‐actPA, shows a notable enhancement in uptake, reaching 4.8 mmol/g, highlighting the effectiveness of structural modulation in optimising NO adsorption and positioning bioNICS‐1 among the highest NO‐uptake MOFs reported under ambient conditions (Figure 1e) [37].

A prominent feature of both materials is the substantial desorption hysteresis, with most NO retained even when the pressure is reduced to 5 mbar. Specifically, retention rates are 96.5% for bioNICS‐1 and 99.0% for bioNICS‐1‐actPA, indicative of strong physisorption and/or chemisorption interactions – an essential factor for controlled NO release.

To investigate the binding dynamics, adsorption kinetics were recorded by dosing 100 mbar of NO gas into evacuated and activated samples at 25°C (Figure 1f). The kinetic data were fitted using two models: the Linear Driving Force (LDF) model (Equation (1) in SI) and the Avrami model (Equation (2) in SI). The LDF model, assuming pseudo‐first‐order kinetics, is typically applied to physisorption‐dominated processes, where the adsorption rate is proportional to the difference between the equilibrium and instantaneous adsorbate concentrations, with diffusion considered uniform across micropores. In contrast, the Avrami model uses a fractional‐order approach, suitable for interpreting multi‐step or complex adsorption mechanisms. Fitted parameters are summarised in Table S3. Pristine bioNICS‐1 exhibits a higher rate constant (*k* = 0.136 min^−1^) in both models, reflecting rapid initial adsorption. The better fit of the kinetic data to the Avrami model (*n* < 0.6) suggests a multi‐step process, where fast physisorption likely facilitates subsequent chemisorption at framework acid sites. This combination of mechanisms results in a relatively high adsorption rate, albeit with a lower overall uptake compared to the modified sample.

In contrast, bioNICS‐1‐actPA displays a lower rate constant (*k* = 0.0145 min^−1^), indicating slower adsorption, likely dominated by chemisorption and diffusion within the functionalised framework. Despite the slower kinetics, this material achieves higher NO uptake at 100 mbar (3.1 mmol g^−1^), consistent with strong interactions between NO and the PA‐modified framework. An Avrami exponent near 1 indicates that adsorption is primarily diffusion‐controlled, with NO readily diffusing into the MOF once adsorption begins, efficiently occupying available sites.

Overall, the kinetics analysis reveals that pristine bioNICS‐1 adsorbs NO faster than its modified analogue despite a lower total capacity, due to the surface accessibility of binding sites. In bioNICS‐1‐actPA, structural modulation introduces additional internal vacancies and functional sites, which slow diffusion and require site reorganisation. These observations indicate a shift in the rate‐determining step from surface‐limited adsorption in pristine bioNICS‐1 to diffusion‐limited adsorption in bioNICS‐1‐actPA.

### 2.2 NO‐Induced Structural Changes

**FIGURE 2:**
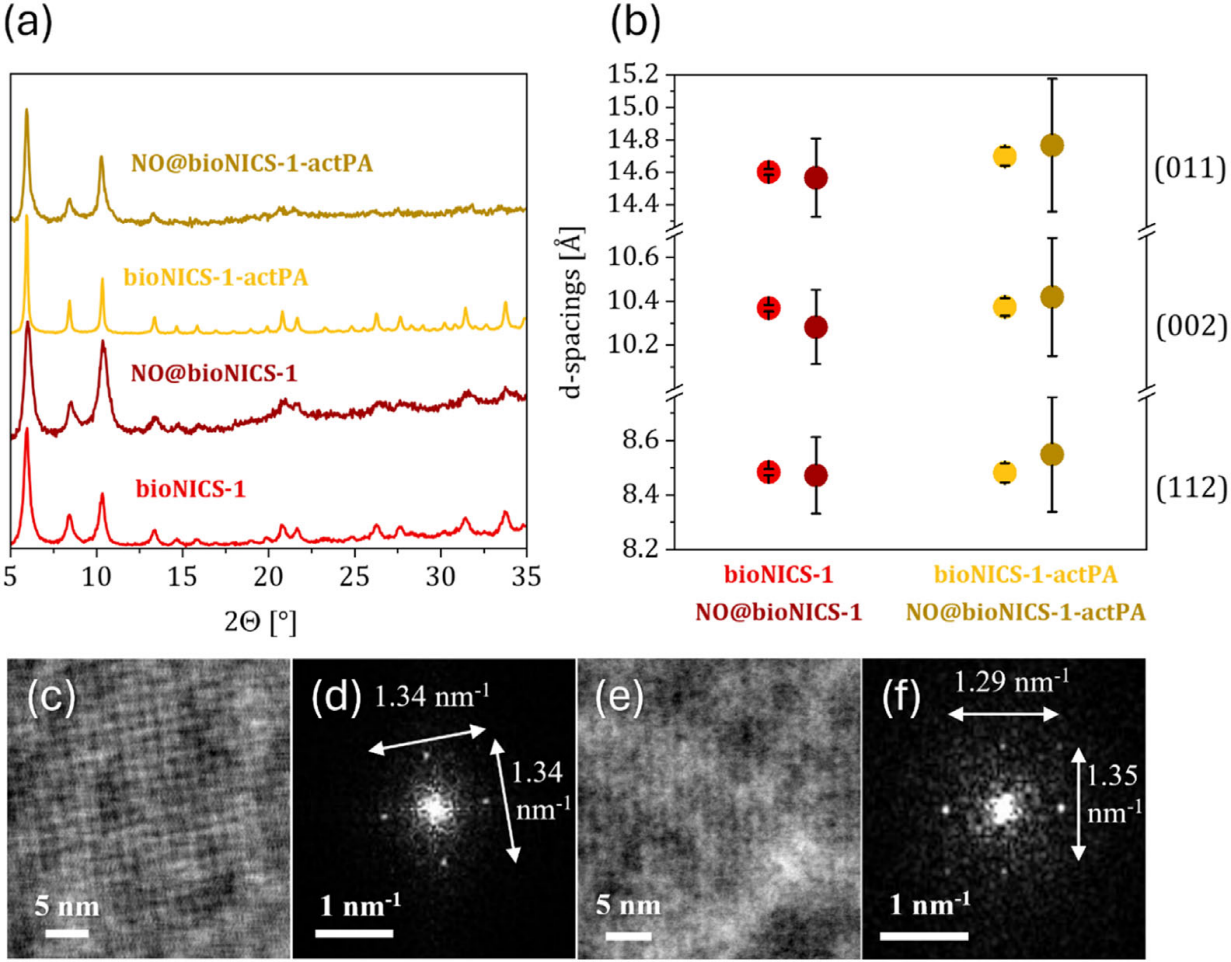
(a) Comparison of XRD patterns for pre‐loaded and NO‐loaded samples. (b) *d*‐spacing values for the preloaded and NO‐loaded materials corresponding to (011), (002), and (112) lattice planes; error bars represent uncertainties obtained from Rietveld refinement of unit cell parameters for each XRD pattern. High‐pass filtered iDPC images of pristine bioNICS‐1 before (c) and after (e) NO treatment. The corresponding fast Fourier transforms (FFTs) from (c) and (e) are given in (d) and (f), respectively. FFT spots representing the (011) reflections from (100) zone axis alignment. The distances of 1.34 nm^−1^ between the reflections (d) correspond to d‐spacings of 1.49 nm. The distances of 1.29 and 1.35 nm^−1^ between the reflections (f) correspond to d‐spacings of 1.55 and 1.48 nm, respectively.

XRD patterns recorded before and after NO loading reveal a pronounced structural response of bioNICS‐1 (Figure 2a). Peak broadening and a substantial reduction in peak intensity indicate partial loss of long‐range order within the framework. Although reliable refinement of unit cell parameters was hindered by the diminished clarity of diffraction peaks, monitoring of the first three reflections, corresponding to the (011), (002), and (112) lattice planes, allows for qualitative assessment of structural changes (Figure 2b).

High‐angle reflections (>12° 2θ) disappear upon NO adsorption, suggesting increased local disorder and disruption of short‐range periodicity, while low‐angle peaks persist, indicating that the overall framework symmetry remains largely intact. Analysis of these low‐angle reflections reveals a slight contraction of the unit cell in pristine bioNICS‐1, reflected as modest decreases in *d*‐spacings, extending toward larger interplanar distances, consistent with heterogenous microstrain. After NO loading, the increased variability in fitted peak positions points to enhanced local distortions. In the PA‐modified analogue, XRD indicates even broader *d*‐spacing distributions, suggesting that structural modification amplifies local flexibility and heterogeneity, which may facilitate NO accommodation within the framework.

To further probe local distortions, high‐resolution STEM analyses were performed on pristine bioNICS‐1 before and after NO exposure. Due to the framework's extreme beam sensitivity, integrated differential phase contrast STEM (iDPC‐STEM) imaging was employed to achieve high contrast and signal‐to‐noise ratios at low electron dose [36]. Corresponding high‐angle annular dark field (HAADF–STEM) images were simultaneously acquired from the same regions (Figure S11).

Before NO loading, iDPC‐STEM measurements reveal a periodicity of 1.49 nm, corresponding to the spacing between repeating lattice planes in the framework (Figure 2c, d). After NO adsorption, the *d*‐spacing increases to 1.55 nm along one direction while remaining nearly unchanged (1.48 nm) in the perpendicular orientation (Figure 2e, f). In some regions, reduced spacing of 1.28 nm was observed, reflecting local contraction along the (011) plane or expansion along the (002) direction (Figure S12). These results reveal that NO induces anisotropic lattice distortions, combining expansion and contraction at the local scale.

Although partial shrinking of MOFs under electron irradiation is common [38], the observed local expansions and anisotropic distortions are consistent with XRD trends and therefore likely reflect intrinsic NO‐induced structural changes rather than beam damage [31].

### 2.3 Mechanistic and Computational Insight Into NO Adsorption and Desorption Process

**FIGURE 3:**
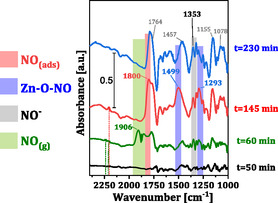
In situ DRIFT spectra of pristine bioNICS‐1 during sequential exposure steps: after degassing in N_2_ (*t* = 50 min), during saturation with 10% NO/N_2_ (*t* = 60 min), after desorption in N_2_ (*t* = 145 min), and after desorption in 3% H_2_O/N_2_ flow (*t* = 230 min). Coloured bars indicate the main vibrational regions associated with different NO species.

To gain an understanding in the structural evolution of bioNICS‐1 during NO adsorption, in situ FTIR/DRIFTS measurements were performed. Both pristine and PA‐modified materials exhibited qualitatively similar spectral features, but for clarity, only the results for pristine bioNICS‐1 are shown in the main text (Figure 3), with corresponding spectra for the modified sample provided in Figures S13–S17. Samples were first evacuated, then exposed to a 10% NO/N_2_, followed by purging with N_2_ – first dry then moist (3% H_2_O). Full spectra and time‐resolved DRIFTS profiles are provided in Figures S14–S17, and reference spectra of the initial, NO‐saturated, and degassed samples are shown in Figure S13.

**FIGURE 4:**
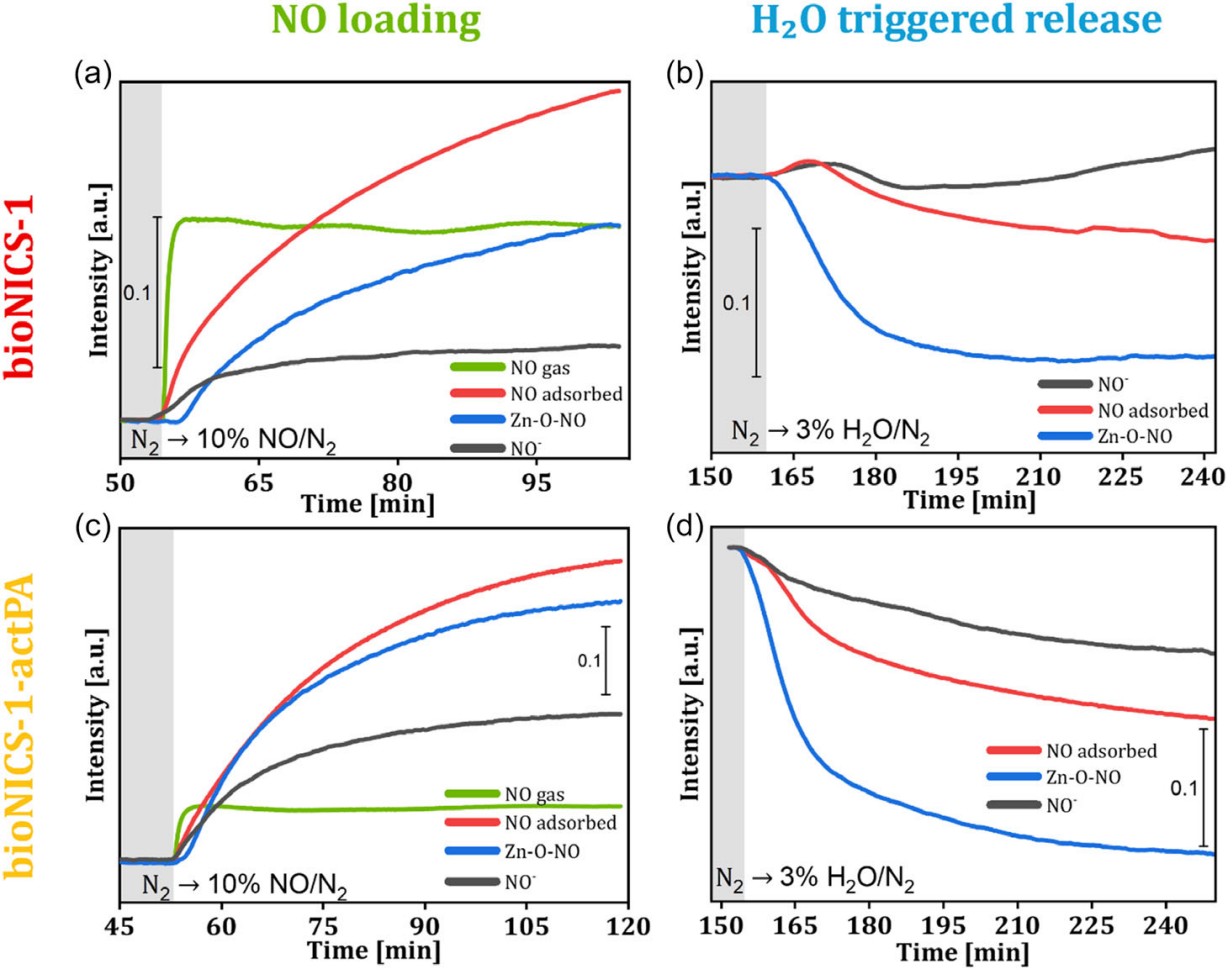
Time‐resolved DRIFT profiles showing the evolution of characteristic bands during NO adsorption (N_2_→10% NO/N_2_ step, a, c) and NOx removal (N_2_ → 3% H_2_O/N_2_ step, b,d) for gas‐phase NO (1905 cm^−1^), physiosorbed NO (1802 cm^−1^), NO^−^ nitrosyl (1353 cm^−1^), and Zn—O—NO (1290 cm^−1^), recorded for both pristine and PA‐modified bioNICS‐1.

The adsorption dynamics were monitored through time‐resolved intensity changes of characteristic NO‐species (Figure 4a and c). Upon introduction of NO, the gas‐phase NO band at 1906 cm^−139^ increased sharply, stabilising within 3 min. Almost immediately, features assigned to physiosorbed NO and linker‐bound nitrosyl (NO^‐^) species appeared, indicating rapid occupation of accessible sites [39, 40]. These linker‐associated sites, likely the hydroxyl groups of the ascorbate moiety, provide electron‐rich environments that stabilise NO via hydrogen bonding and weak donor–acceptor interactions.

In general, NO interacting with redox‐active or polar sites can form a variety of N_*x*_O_*y*_ species due to its ability to engage in both σ‐ and π‐ bonding [39]. Negatively charged nitrosyl species preferentially interact with cationic or acidic sites within the material, such as exposed metal centres or polar functional groups on the organic linker. These interactions stabilise the adsorbed nitrosyls through electrostatic attraction or hydrogen bonding. Redox‐active or polar moieties, including ascorbate groups, may further enhance stabilisation via electron donation or additional hydrogen‐bonding interactions, increasing overall adsorption affinity. The band at 1353 cm^−1^, corresponding to NO^−^ coordinated to linker or acidic sites [40], gradually increased and reached steady state after ∼25 min, whereas the physiosorbed NO continued to rise over 65 min. This slower evolution reflects diffusion‐limited uptake within the micropores (Figure S6), consistent with the previously observed kinetic data. Adsorption at Zn—O node sites, initiated only after a 2–3 min delay, suggesting that minor local structural rearrangements are required to activate these metal sites. Concurrent spectral changes in hydroxyl, C—H, and C=O stretching regions (Figure S18) indicate partial linker displacement or coordination reorganisation upon NO binding. The Zn—O—NO bands reached only partial saturation even after 65 min, reflecting limited accessibility and slower kinetics at these nodes.

Overall, the data indicate sequential NO adsorption: initial binding to the linker −OH groups form NO^−^ species, which induces local structural modifications that subsequently expose additional Zn—O sites for chemisorption.

During subsequent desorption under wet N_2_ flow, the NO^‐^ band intensity remained nearly constant for pristine bioNICS‐1 but decreased gradually in bioNICS‐1‐actPA, indicating lightly weaker retention in the modulated framework. In contrast, the Zn—O—NO band decayed rapidly, reaching steady state within ∼70 min, consistent with facile displacement by water molecules. A transient increase in the NO^‐^ band during early adsorption may reflect migration of NO from Zn—O nodes to linker sites or overlap with framework vibrations. Based on these kinetics, the apparent NO binding strength follows: Zn—O—NO > linker‐NO > physiosorbed NO, while release rates are modulated by water competition.

**FIGURE 5:**
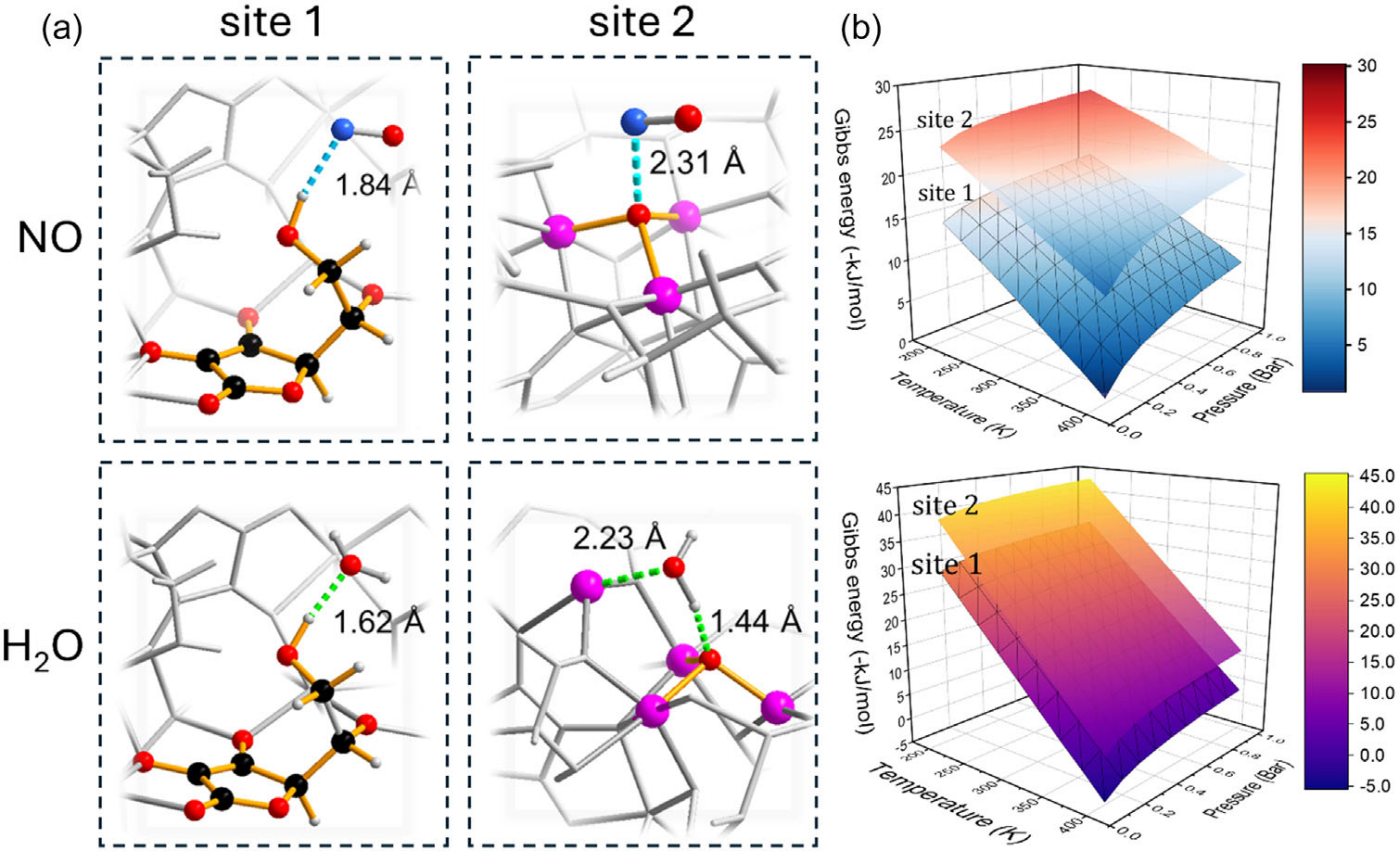
DFT‐optimised structures and thermodynamic profiles for NO and H_2_O adsorption on bioNICS‐1. (a) Optimised structures at linker‐associated (site 1) and Zn‐based inorganic (site 2) positions, for NO and H_2_O adsorption at the corresponding sites, respectively. NO interacts via hydrogen bonding with the ascorbate linker (O—H···N—O, 1.84 Å) or through coordination of the N atom to the μ_3_‐oxide bridge (N···O=2.31 Å). In contrast, H_2_O forms stronger interactions through O—H···O (1.62 Å) and μ_3_−O ···Zn (1.44 Å) bonds, indicating its potential to replace pre‐adsorbed NO. (b) Gibbs free energy (Δ*G*) maps as a function of temperature and pressure for NO and H_2_O adsorption show both processes are exergonic under ambient conditions, with a stronger thermodynamic driving force for H_2_O binding compared to NO.

Computational insights further support this sequential adsorption mechanism. Ab initio density functional theory (DFT) calculations identify two distinct adsorption sites for NO (Figure 5a): (1) Linker‐associated site 1, where NO interacts with hydroxyl groups of the ascorbate linker, forming a hydrogen‐bonding motif, O—H···N—O···H—C. This localised polar environment stabilises NO with the calculated adsorption energy of −32 kJmol^−1^, consistent with the rapid formation of NO^‐^ species observed in DRIFTS; (2) Zn‐based node site 2, NO coordinates to the µ_3_‐oxide atom, bridging three ZnO_6_ octahedra. This configuration represents metal‐associated adsorption with slightly stronger energy (−41 kJmol^−1^). The delayed appearance and slower decay of the Zn—O—NO band in DRIFTS confirm this assignment, suggesting that minor relaxation of the Zn—O cluster is required prior to NO coordination.

Although both adsorption modes are exothermic, Gibbs free energy (Δ*G* < 0 under ambient conditions) suggest that site 2 is slightly more thermodynamically favourable, explaining the sequential binding: initial rapid adsorption at linker −OH groups, followed by slower coordination to the μ_3_‐oxide centres. (Figure 5b and S20).

DFT modelling also reveals that H_2_O binds more strongly than NO at both sites (−79 kJ/mol at site 1, –84 kJ/mol at site 2), providing a mechanistic basis for water‐triggered NO release. Water preferentially coordinates to the Zn—O cluster at site 2, displacing NO, while at site 1, hydrogen bonding with hydroxyl‐rich linkers facilitates NO removal. This sequential desorption aligns with the experimental DRIFTS profiles, demonstrating that competitive adsorption governs humidity‐triggered NO release.

In summary, the combined DRIFTS and DFT results reveal a dual‐site, sequential NO adsorption mechanism in bioNICS‐1. Initially, NO rapidly interacts with hydroxyl groups of the ascorbate linker, forming weakly bound NO^‐^‐type species stabilised by hydrogen bonding and polar interactions. This initial adsorption step induces local structural rearrangements that facilitate subsequent access to Zn‐based sites. In a second, slower step, NO coordinates to μ_3_—O—Zn nodes, resulting in more strongly bound species and contributing to the observed adsorption hysteresis and diffusion‐limited kinetics. Water molecules act as the dominant trigger for NO desorption, preferentially displacing NO from Zn‐O sites, followed by the removal of linker‐associated NO, explaining the site‐dependent and stepwise release behaviour.

### 2.4 NO Release Studies in Liquid Environment

To continue the exploration into water‐triggered NO release from bioNICS‐1 (and bioNICS‐1‐actPA), oxyhaemoglobin [41] and Griess [42] assay were performed, which represent more real‐environment conditions and are already well established methods for gathering insight into NO release from zeolites and MOFs [43].

**FIGURE 6:**
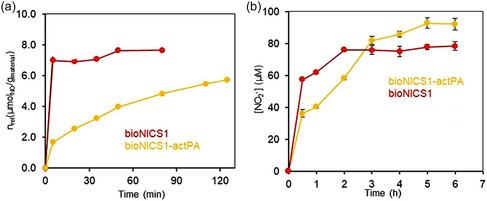
Oxyhaemoglobin (a) and Griess (b) to test the release of NO from bioNICS‐1 and bioNICS‐1‐actPA loaded with NO. Data are presented as ± standard deviation (SD) based on three independent measurements.

The transformation of oxyhaemoglobin to methaemoglobin observed for both samples loaded with NO indicates that the NO is delivered at a fast rate from the materials (Figure 6a, S21 and S22). This type of assay is mainly informative of the initial release kinetics at very low concentrations and not so significant when a plateau is attained (as a complete consumption of oxyhaemoglobin may affect the results at this stage). Pristine bioNICS‐1 achieves almost a complete release within the first 10 min, whereas bioNICS‐1‐actPA releases NO more gradually for approx. 2 h.

It is known form our previous work [31] that in biologically relevant media (such as water, saline solution and phosphate buffer solution) bioNICS‐1 forms a shell of Zn‐oxalate and Zn‐phosphate domains which subsequently erodes [31]. This is shown as a slow release of Zn^2+^ to the media, and it is most likely the reason for the rapid initial release of NO from unmodified bioNICS‐1 in aqueous media. It also suggests that the adsorption mechanism in bioNICS‐1 is dominated by physisorption, which involves weaker, reversible interactions that are more responsive to external stimuli like humidity, allowing NO to desorb quickly when exposed to water media.

The Griess assay results (Figure 6b) confirm to some extent that the materials have a fast initial release of NO, since the first measurements at 30 min contact time already indicate a significant increase of the nitrite concentration in solution formed by the NO release (57 and 36 µM, for bioNICS‐1 and bioNICS‐1‐actPA). However, it distinguishes the materials showing that the bioNICS1‐actPA can deliver more NO with a slightly slower rate than the unmodified bioNICS‐1. This indicates that the activation with PA introduces new active sites for NO binding (in agreement with the results above, Figure 1b) with slower NO release. Longer and slower release of NO for bioNICS‐1‐actPA (>120 min in OxyHb and 6 h in Griess assay) can be attributed to stronger NO–framework interactions (Figure 1b), and that adsorption of NO is dominated by chemisorption. Analysis of kinetics suggests a process largely controlled by diffusion, typical of chemisorption‐dominated mechanisms. Slower release pattern of bioNICS‐1‐actPA, even under water exposure, aligns with controlled diffusion seen in this sample.

### 2.5 Future Prospect

The intended application for this material is in biomedicine, specifically for delivering therapeutically relevant amounts of NO. To evaluate the effect of the un‐loaded material on cells, a preliminary test of possible toxicity to skin cells was performed. Due to its partial solubility in cell culture media (Table S4), bioNICS‐1 exhibits concentration‐dependent toxicity, consistent with the known cytotoxic effects of Zn^2+^ under in vitro conditions [44]. Nevertheless, with an IC50 of 48.21–53.15 µg mL^−1^ (Figure S23), bioNICS‐1 falls within the acceptable toxicological range and therefore remains a promising candidate for further biological evaluation.

Unlike previously described MOFs for linker‐based NO adsorption [15, 16, 17, 18, 19], there is no need for amino‐functionalization of bioNICS1 linker (ascorbic acid) or CUS site. A common preparation of MOFs for gas adsorption is temperature‐induced activation [45], which in the case of bioNICS1 might not even be necessary, since the NO adsorption on the linker effectively activates the CUS. This carries a great scale‐up and health and safety advantage.

When compared with the current benchmark material (in regards to duration of NO release), MIP‐210 [46], which is capable of releasing NO for ≥70, the 2 h release profile of bioNICS‐1 (and bioNICS‐1‐actPA) appears relatively short. However, this characteristic can be advantageous. Moving beyond a 'one for all' strategy, where a single material is expected to meet all therapeutic demands, toward an 'all for one' approach, in which multiple tailored materials are combined to address a specific clinical setting, new opportunities emerge. For instance, in wound‐healing applications where tissue regeneration is impaired (e.g., diabetic wounds), bioNICS‐1 and MIP‐210 could be co‐formulated. A short, initial NO burst from bioNICS‐1 could help reduce the bacterial burden on the wound surface, while prolonged release from MIP‐210 would sustain NO delivery throughout the healing process.

## 3 Conclusions

This study identifies bioNICS‐1, a Zn–ascorbate bioMOF, as a promising platform for NO capture and controlled release, and introduces a new design strategy based on radical‐scavenging linkers as active adsorption sites. By integrating adsorption measurements, kinetic modelling, in situ spectroscopy, microscopy, and DFT calculations, we demonstrate that NO binding proceeds through a dual‐site, sequential mechanism, involving rapid interaction with ascorbate linker –OH groups followed by slower coordination to Zn–O nodes. This provides the first experimental evidence of a linker‐driven NO sorption pathway in MOFs.

Structural modification via PA introduces defects that enhance porosity and generate additional binding environments, leading to an increase in NO uptake (up to 4.8 mmol g^−1^) and a transition toward stronger, chemisorption‐dominated interactions. As a result, adsorption kinetics and release profiles can be systematically tuned: pristine bioNICS‐1 exhibits fast uptake and burst release, whereas the modified analogue enables slower, more sustained NO delivery. The pronounced adsorption–desorption hysteresis and in situ spectroscopic data further confirm strong, multi‐step host–guest interactions.

Both experimental and computational results show that water acts as the key trigger for NO release, displacing NO from both metal and linker sites. This enables controlled release under physiologically relevant conditions, which is supported by liquid‐phase assays demonstrating distinct and tuneable release behaviours for the two materials.

Overall, this work delivers three main contributions: (i) it validates radical‐scavenging linkers as functional components for gas adsorption, (ii) it reveals a cooperative linker–metal adsorption mechanism governing NO storage and release, and (iii) it demonstrates that defect engineering can indeed tune performance in bioMOFs. These findings position bioNICS‐1 as a promising candidate for therapeutic gas delivery and provide a foundation for the rational design of next‐generation MOFs for biomedical applications.

## 4 Materials and Methods

### 4.1 Sample Preparation Procedures and Materials

Synthesis protocols and activation procedures are thoroughly described in Supplementary information.

### 4.2 Characterisation Methods

X‐ray powder diffraction data of the samples were collected on a PANalytical X’Pert PRO high‐resolution diffractometer (Malvern Panalytical, Almelo, The Netherlands) with CuKa radiation (*λ *= 1.5406 Å) in the range from 5° to 60° (2θ) with a step of 0.034° per 100 s using a fully opened 100 channel X’Celerator detector. For the purposes of Rietveld refinement of the crystal structure model, the XRD powder data were collected on the same equipment using a transmission mode in the range from 5° to 35° 2Θ with a step of 0.016°/300 s. The diffractograms were analysed, and the particle size was calculated using the Sherrer equation with the HighScore Plus 4.9 program package (Malvern Panalytical B.V.).

Thermal analysis (TG/DTG) was performed on a Q5000 IR thermogravimeter (TA Instruments, Inc., New Castle, DA, USA). The measurements were carried out in an air flow of 10 mL/min, by heating samples from 25°C to 700°C at a rate of 10°C/min. The temperature‐programmed X‐ray powder diffraction pattern of samples was recorded also on the PANalyticalX’Pert PRO diffractometer, additionally equipped with a high temperature sample cell, from room temperature to 500°C in steps of 50°C in static air.

Visualisation of the samples was observed by scanning electron microscopy measurements (SEM) on a Zeiss Supra 3VP field‐emission gun (FEG) microscope (Carl Zeiss AG, Oberkochen, Germany). Elemental analysis was performed by energy dispersive X‐ray analysis (EDAX) with an INCA Energy system attached to the above‐described microscope and by Perkin Elmer 2400 Series II CHNS analyser (Perkin Elmer, Waltham, MA, USA).

N_2_ sorption isotherms measurements were performed on Quantachrome AUTOSORB iQ3 (Anton Paar, Graz, Austria). The specific surface areas were determined by the Brunauer–Emmett–Teller (BET) method based on the N_2_ sorption isotherms measured at 77 K in a *p*/*p*_0_ relative pressure range between 4 × 10^−2^ and 6 × 10^−3^, selected according to the Roquerol plots. Before the measurement, samples were activated under a vacuum at 150°C for 12 h. Pore size distribution analysis (PSD) was performed using the NLDFT procedure based on the adsorption data.

Acid sites were quantitatively evaluated using ammonia adsorption/adsorption experiments on a dynamic vapour sorption analyser (DVS, Surface Measurement Systems Ltd., London, UK). Prior to the measurements, the samples were outgassed at 150°C for 12 h. Ammonia dynamic adsorption was performed on activated materials using 10% NH_3_ in an argon flow of 5 mL/min, gradually increasing pressure from vacuum to 1 bar with a step of 100 mbarm measuring equilibrium mass gain for each pressure step. The desorption process, using steps of 200 mbar, was followed by heating up to 150°C with a ramp of 10°C/min.

To analyse the sorption capacities and monitor the adsorption kinetics of NO binding on bioNICS‐1, gravimetric analysis was conducted on a DVS (Surface Measurement Systems Ltd., London, UK), where the change in sample mass during controlled dosing of the adsorbate was directly monitored. Prior to NO loading, samples were degassed at 150°C for 12 h. Dynamic NO sorption was carried out using 99.5% NO at a flow rate of 5 mL/min, with pressure gradually increasing from vacuum to 1 bar at intervals of 100 mbar. Measurements of equilibrium mass uptake were performed for each pressure level. Desorption was conducted in steps of 200 mbar at a temperature of 25°C.

The samples were kept inside the glove box under a continuous Argon atmosphere until the experiment. They were prepared inside the glove box by mixing the powders with ethanol. Just before the experiment, the samples were taken out of the glove box, sonicated for a few minutes, and then drop‐cast onto copper TEM grids coated with amorphous carbon. A Thermo Fisher Scientific Titan Cubed electron microscope (TEM) operating at 300 kV was utilised for energy dispersive X‐ray spectroscopy (EDX) measurements, and the simultaneous acquisition of high‐angle annular dark field scanning transmission electron microscopy (HAADF–STEM) and iDPC images. A probe convergence semi‐angle of 17 mrad and a camera length of 230 mm were used, yielding inner and outer collection semi‐angles of 26–154 mrad for the HAADF detector and 6–24 mrad for the DF4 detector. iDPC images were high‐pass filtered (sigma of 80 pixels) to enhance image contrast by reducing low‐frequency components.

To understand the interaction of NH_3_ and NO with bioNICS‐1, quantum chemical calculations at the DFT level were performed using the VASP 6.3.1 software. As a good compromise between the chemical accuracy and computational cost, a well‐known PBE function was employed. An energy cut‐off of 500 eV sufficed for well‐converged results. Due to the size of the unit cell, the reciprocal space was sampled at a single point (gamma). For the calculations of NO, spin‐polarised calculations were required to account for the non‐paired electron. NH_3_ and bioNICS‐1 have closed electronic shells. Calculations were performed using a Gaussian smearing of 0.03 eV.

Initially, a full geometric and structural optimisation was performed, where the size of the unit cell and the atomic positions in bioNICS‐1 were allowed to change, revealing the lattice parameters. The force threshold for optimisation was set at 0.01 eV/Å. Having identified the optimum dimension of the unit cell, it was then kept fixed as several possible sites for NO and NH_3_ adsorption were probed, and two distinct (accounting for symmetry) were identified. The energy of adsorption was calculated as

$$E_{ads}=E_{\text{bioNICS}1+\text{adsorbate}}-E_{\text{bioNICS}1}-E_{\text{gaseous}}$$

where $E_{\text{bioNICS}1+\text{adsorbate}}$ is the electronic energy of bioNICS‐1 with the adsorbate, $E_{\text{bioNICS}1}$ is the energy of empty bioNICS‐1 and $E_{\text{gaseous}}$ is the energy of the unperturbed adsorbate molecule in vacuum. In all instances, a Grimme D3 correction to describe the van der Waals interactions was applied since vanilla DFT severely underestimates them.

To account for the temperature effects, the Gibbs free energies of the adsorption were also calculated. For bioNICS‐1, we can assume that G=E, as we have ΔS≈0 and ΔV≈0 upon adsorption. For the adsorbates, an ideal gas approximation is used in the gaseous phase (vibrationa, rotational, translational degrees of freedom) and the harmonic approximation is used in the adsorbed state, including the vibrational degrees of freedom only.

To study the geometric parameters of the structure, a Monte Carlo simulation with 200.000 hits was performed on the DFT‐optimised structure. Using Zeo++, which uses the Voronoi decomposition, we have calculated the pore diameter, surface area, accessible volume, pore‐size distribution, stochastic ray tracing and probe‐occupiable volume for bioNICS‐1. The accessible surface area was evaluated for probes of various radii (0.5–3.5 Å).

Time‐resolved DRIFT spectroscopy was performed on a Perkin Elmer model Frontier spectrometer equipped with a DiffusIR reaction chamber from Pike Scientific. Spectra were recorded using Timebase software and a liquid nitrogen‐cooled MCT detector. The analysed spectral range was between 1000 and 4000 cm^−1^, spectral resolution of 4 cm^−1^ and four accumulations per scan, allowing us to achieve a time resolution of about 15 s. For the analysis, about 10 mg of powdered sample was positioned inside the ceramic sample holder and sealed with a KBr window. The samples were degassed overnight in a vacuum (10^−5^ mbar, pump model Hi cube by Pfeiffer vacuum) at 120°C. After degassing, the cell was pressurised with nitrogen (purity 5.0) to ambient pressure, and flow was maintained at 10 mL/min using Brooks electronic mass flow controllers (model 5850). After cooling the sample to 25°C in N_2_ flow, the background was recorded with a degassed sample, and spectral recording was initiated. After 15 min in N_2_ flow, the sample was saturated with NO gas (1 mL/min, mixed with 10 ml/min N_2_ flow) at 25°C for about 30 min. After NO saturation, the sample was flushed in N_2_ flow until all gas phase NO was removed, and the NO desorption was initiated by moist N_2_. Nitrogen was saturated with water vapour at 23°C by passing the flow through a saturator.

NO release studies in the liquid phase were conducted using the oxyhaemoglobin assay as described by Feelisch et al. [41], which is highly specific for the detection and quantification of NO under aerobic conditions. This method is based on the basic reaction of oxyhaemoglobin (oxyHb) with NO, forming methaemoglobin (metHb) and nitrate.

$$\mathrm{oxyHb}\;+\;\mathrm{NO}\;\to \;\mathrm{metHb}\;+\;{\mathrm{NO}}^{3‐}$$

Polytetrafluoroethylene (PTFE) was added to activated bioNICS‐1 samples at a ratio of 75% (sample): 25% (PTFE) (w/w). The mixture was compressed into discs (under 8 tons for 30 s) to avoid sample dispersion in the liquid phase. Discs weighing approximately 5 mg were inserted into a cell with a vacuum valve, followed by sample degassing for 2 h at 150°C, with the vacuum greater than 10^−2^ Pa.

After degassing, NO was introduced into the cell under a pressure of 80 kPa, the condition was maintained for 3 days by closing the valve and removing the cell from the vacuum line. After loading NO, the remaining NO in the cell was evacuated, and was immediately filled with helium to atmospheric pressure. The cell remained closed until the start of the oxyhaemoglobin test. Oxyhaemoglobin solution (oxyHb) was prepared using the previously described method [42]. Briefly, 20 mg of lyophilised human haemoglobin was dissolved in 1 mL of buffer solution, and sodium dithionite was added to ensure complete reduction of haemoglobin. Sephadex G‐25 column was used for purification and removal of salts from the obtained oxyHb solution.

The NO release experiment was conducted at room temperature using a UV/vis spectrophotometer (Genesys 10S, Thermo Scientific) and quartz cuvettes with a volume of 3 mL. The first spectrum measurement was performed with an oxyHb solution (1 μM) without the sample, serving as a reference. Subsequently, the cell containing the NO‐loaded sample was opened, and the sample was immediately added to the cuvette with the oxyHb solution. Spectra were recorded every 15 min for 3 h. The kinetic profile of NO release was calculated according to the protocol presented in the seminal work of Feelisch et al. [41].

NO release studies in the liquid phase were also conducted using the Griess assay.

NO loading in BioNICS (powder):

For the Griess assay, 5 mg of the BioNICS powder sample was measured in a glass cell, which was then introduced into the adsorption line and put under high vacuum via a high vacuum pump system composed of a turbomolecular pump and a diaphragm pump (Pfeiffer Vacuum). The sample was then outgassed for 2 h at 150°C, after which NO was introduced to the system until a pressure of 80 kPa was achieved and left to adsorb for 3 days. After the NO loading period, the remaining NO inside the cell was evacuated under vacuum and immediately filled with helium up to atmospheric pressure to avoid premature release.

NO release:

The NO released over time from the material was quantified indirectly through its stable decomposition product, nitrite (NO_2_^−^), which accumulated in the liquid medium and was measured using the Griess method. The loaded material was incubated in a phosphate buffer (pH = 7.4) at 450 µg/mL. Samples were taken from the liquid medium after 30 min, 1 h, and then every hour until the sixth hour. The samples were then incubated with the Griess reagent (0.1% naphthylethylenediamine dihydrochloride and 1% sulphanilamide in 5% phosphoric acid), producing a chromophoric azo product through the reaction with nitrite. The absorbance of this species was measured at 548 nm using a microplate reader (Tecan, A‐5082 Sunrise Remote), and the values were converted into equivalent nitrite concentrations using a calibration curve prepared with sodium nitrite solution and the Griess reagent, following the same procedure used for the samples. The calibration curve is presented in Figure S22.

### 4.3 In Vitro Cytotoxicity

#### 4.3.1 Cell Culture

HaCaT cells, a spontaneously immortalised keratinocyte cell line derived from adult human skin (CLS 300 493; CLS Cell Lines Service GmbH), were cultured in DMEM medium Dulbecco's Modified Eagle Medium (DMEM medium; Sigma) with 10% Fetal bovine serum (FBS; Sigma) under standard conditions (37°C, 5% CO_2_). Cells were subcultured using trypsin (Sigma) before reaching confluence. Cells were used within 10 consecutive passages.

#### 4.3.2 Stability of bioNICS1 in Cell Medium (in Culture Medium)

The stability of the activated material – bioNICS1 in the cell medium (culture medium) was tested under incubation conditions (37°C, 5% CO_2_) for 24 and 48 h. We tested the highest concentration used – 900 µg/mL. The concentration of released Zn^2+^ was determined using the ICP‐OES method.

#### 4.3.3 Cytotoxicity Test

Particle toxicity was assessed by monitoring changes in the metabolic activity of HaCaT cells 24 and 48 h after adding the MOF particle suspension. The day before the experiment, cells were seeded onto a white 96‐well microplate (Costar) at a density of 20,000 cells per well in 100 µL of medium. The next day, cells were washed twice with DMEM medium without supplements and then treated with the test suspension of MOF microparticles in a concentration range of 3.5–900 µg/mL (material suspended in colourless DMEM medium) and incubated for 24 or 48 h. After incubation, the metabolic activity of cells was determined using the PrestoBlueTM Cell Viability Reagent (Thermo Fischer Scientific), according to the manufacturer's instructions. Substrate conversion was determined by fluorescence measurement. The relative metabolic activity of exposed cells to particles was calculated as the percentage of metabolic activity of non‐exposed control cells.

## Funding

This study was supported by Javna Agencija za Raziskovalno Dejavnost RS (P1−0021, P2−0152, P1−0418, J1−50020, I0−0039, J2−4424, N2−0310, J7−4638, J2−4441, N2−0265), Fundação para a Ciência e a Tecnologia (PIDDAC ‐ UIDB/00100/2025(CQE), UIDP/00100/2025, LA/P/0056/2020(IMS), UIDB/04028/2025, UIDP/04028/2020 (CERENA), 2022.05605.PTDC), Flemish Fund for Scientific Research (FWO Vlaanderen) (1181122N, 1181124N).

## Conflicts of Interest

The authors declare no conflicts of interest.

## Supporting information

Supplementary Material

## Data Availability

The data that support the findings of this study are available from the corresponding author upon reasonable request.
