# Supplementary material for: Mechanistic Insights Into Nitric Oxide Capture and Release in a Radical‐Scavenging Zinc Ascorbate Metal–Organic Framework
Source: Small Sci. 2026 May 18;6(5):e70312. doi: 10.1002/smsc.70312 (PMC13185873; doi:10.1002/smsc.70312)
Supplement: Supplementary file 1 — Supplementary Material [file SMSC-6-e70312-s001.pdf]

SUPPORTING INFORMATION

Mechanistic Insights into Nitric Oxide Capture and Release in a Radical-Scavenging Zinc-Ascorbate Metal-Organic Framework

Contents

1. **Overview of some radical scavengers** 3
2. **Sample preparation procedures and materials** 4
3. **Characterisation methods** 5
4. **Structural examination** 10
5. **NO sorption mechanism** 17
6. **High-angle annular dark field scanning transmission electron microscopy** 18
7. **FTIR/DRIFTS *in situ* measurements** 19
8. **Oxyhaemoglobin and Griess assay** 24
9. **Preliminary *in vitro* study** 25
10. **Literature** 26

1. Overview of some radical scavengers

TABLE S1: Examples of radical scavengers

<table>
<tr><th colspan="2">RADICAL SCAVENGERS</th></tr>
<tr><th>endogenous</th><th>exogenous</th></tr>
<tr><td>Enzymatic: Superoxide dismutase (SOD), Catalase (CAT) and glutathione peroxidase (GPx), Glutathione reductase, Glucose-6-phosphate dehydrogenase, melatonin</td><td rowspan="2">Vitamins (vit. C & E), trace elements (Zn & Se), Carotenoids (β-carotene, lycopene, lutein), phenolic acids (chlorogenic acids, gallic acid, caffeic acid …), Polyphenols (flavonoid: quercetin, ellagic acid, hesperetin, luteolin … and Non-flavonoid: genistein, curcumin, resveratrol …), metalloporphyrins (methylcobalamin = vit. B12, Mn(III) tetrakis(4-benzoic acid) porphyrin (MnTBAP))</td></tr>
<tr><td>Non-enzymatic: Glutathione (GSH), uric acid, lipoic acid, NADPH, coenzyme Q, albumin, metalloporphyrins (haemoglobin, myoglobin, bilirubin)</td></tr>
</table>

2. Sample preparation procedures and materials

<u>Unmodified procedure of bioNICS-1</u>

BioNICS-1 was prepared using already established synthesis protocol[1,2]. Typically, 0.752 g of zinc(II) acetate dehydrate (Sigma Aldrich, St. Louis, MO, USA) and 0.3 g of L-ascorbic acid (Sigma-Aldrich, St. Louis, MO, USA) are added to 9.97 ml of ethanol (Sigma-Aldrich, St. Louis, MO, USA)*****. The reaction mixture was then heated in a conventional (120 °C for 24h) or microwave-assisted (600 W at 120 °C for 2 h) way in Teflon-lined autoclaves. Filtered and air-dried samples were then activated with a simple "wash", which was conducted in a round bottom flask with reflux cooling on an oil bath in absolute EtOH******, at 60 °C, under constant stirring for approximately 12 h. Following the filtration, the product was dried in a vacuum oven at 100 °C for 3 h. FTIR spectroscopy confirmed the successful incorporation of ascorbate into the Zn-based framework. Characteristic vibrational bands of free ascorbic acid—such as the broad O–H stretch (~3200–3600 cm^{-1}) and sharp carbonyl-related modes (~1750–1650 cm^{-1})—were either significantly shifted or absent in the product spectrum. This indicates coordination of the ligand and the absence of unreacted ascorbic acid in the final material (Figure S24).

<u>***** *in situ* modification:</u>

After mixing the reaction mixture described above, add propionic acid (6 mol) (Sigma-Aldrich, St. Louis, MO, USA, 99.5%). Proceed with the microwave-assisted synthesis and activation as described above.

<u>****** post-synthesis modification:</u>

When activating the conventionally synthesised product, suspend unmodified material in absolute EtOH add 10% of propionic acid and proceed as described above.

Table S2: List of samples – samples in bold are studied and presented in the article.

	Sample name	Heating source (synthesis)	Modulator	Duration of the synthesis
Unmodified procedure	**bioNICS-1**	Conventional oven	none	1 day
	bioNICS-1-MW	Microwave oven	none	2h
in situ modification	bioNICS-1-synPA	Microwave oven	Propionic acid	2h
post-synthesis modification	**bioNICS-1-actPA**	Conventional oven	Propionic acid	1 day

bioNICS-1 structure is obtained and retained after in situ and post-synthesis modification (Fig. S1). The dimensionality of infinite 3D inorganic building units and the rigidity of the structure are the most likely reasons for retaining the crystallinity even in harsh environments.

3. Characterisation methods

X-ray powder diffraction data of the samples were collected on a PANalytical X'Pert PRO high-resolution diffractometer (Malvern Panalytical, Almelo, The Netherlands) with CuKa radiation (λ = 1.5406 Å) in the range from 5 to 60° (2θ) with the step of 0.034° per 100 s using fully opened 100 channel X'Celerator detector. For the purposes of Rietveld refinement of the crystal structure model, the XRD powder data were collected on the same equipment using a transmission mode in the range from 5 to 35° 2Θ with the step of 0.016°/300 s. The diffractograms were analysed and the particle size was calculated using the Sherrer equation with the HighScore Plus 4.9 program package (Malvern Panalytical B.V.).

Thermal analysis (TG/DTG) was performed on a Q5000 IR thermogravimeter (TA Instruments, Inc., New Castle, DA, USA). The measurements were carried out in air flow of 10 ml/min, by heating samples from 25 °C to 700 °C at a rate of 10 °C/min. The temperature-programmed X-ray powder diffraction pattern of samples was recorded also on the PANalyticalX'Pert PRO diffractometer, additionally equipped with a high temperature sample cell, from room temperature to 500 °C in steps of 50 °C in static air.

Visualization of the samples was observed by scanning electron microscopy measurements (SEM) on a Zeiss Supra™ 3VP field-emission gun (FEG) microscope (Carl Zeiss AG, Oberkochen, Germany). Elemental analysis was performed by energy dispersive X-ray analysis (EDAX) with an INCA Energy system attached to the above-described microscope and by Perkin Elmer 2400 Series II CHNS analyser (Perkin Elmer, Waltham, MA, USA).

N_2 sorption isotherms measurements were performed on Quantachrome AUTOSORB iQ3 (Anton Paar, Graz, Austria). The specific surface areas were determined by Brunauer–Emmett–Teller (BET) method based on the N_2 sorption isotherms measured at 77 K in p/p_0 relative pressure range between 4×10^{-2} and 6×10^{-3}, selected according to Roquerol plots. Before the measurement, samples were activated under a vacuum at 150 °C for 12 h. Pore size distribution analysis (PSD) was performed using the NLDFT procedure based on the adsorption data.

Acid sites were quantitatively evaluated using ammonia adsorption/adsorption experiments on a dynamic vapor sorption analyzer (DVS, Surface Measurement Systems Ltd., London, UK). Prior to the measurements, the samples were outgassed at 150 °C for 12 h. Ammonia dynamic adsorption was

performed on activated materials using 10% NH_3 in an argon flow of 5 ml/min, gradually increasing pressure from vacuum to 1 bar with the step of 100 mbar measuring equilibrium mass gain for each pressure step. The desorption process, using steps of 200 mbars, was followed by heating up to 150 °C with a ramp of 10 °C/min.

To analyse the sorption capacities and monitor the adsorption kinetics of NO binding on bioNICS-1, gravimetric analysis was conducted on a dynamic vapor sorption analyzer (DVS, Surface Measurement Systems Ltd., London, UK), where the change in sample mass during controlled dosing of the adsorbate was directly monitored. Prior to NO loading, samples were degassed at 150°C for 12 hours. Dynamic NO sorption was carried out using 99.5% NO at a flow rate of 5 mL/min, with pressure gradually increasing from vacuum to 1 bar at intervals of 100 mbar. Measurements of equilibrium mass uptake were performed for each pressure level. Desorption was conducted in steps of 200 mbar at a temperature of 25°C.

The samples were kept inside the glove box under a continuous Argon atmosphere until the experiment. They were prepared inside the glove box by mixing the powders with ethanol. Just before the experiment, the samples were taken out of the glove box, sonicated for a few minutes, and then drop-cast onto copper TEM grids coated with amorphous carbon. A Thermo Fisher Scientific Titan Cubed electron microscope (TEM) operating at 300 kV was utilized for energy dispersive X-ray spectroscopy (EDX) measurements, and the simultaneous acquisition of high-angle annular dark field scanning transmission electron microscopy (HAADF-STEM) and integrated differential phase contrast (iDPC) images. A probe convergence semi-angle of 17 mrad and a camera length of 230 mm were used, yielding inner and outer collection semi-angles of 26-154 mrad for the HAADF detector and 6-24 mrad for the DF4 detector. iDPC images were high-pass filtered (sigma of 80 pixels) to enhance image contrast by reducing low-frequency components.

To understand the interaction of NH_3 and NO with bioNICS-1, quantum chemical calculations at the density functional theory (DFT) level were performed using the VASP 6.3.1 software. As a good compromise between the chemical accuracy and computational cost, a well-known PBE function was employed. An energy cut-off of 500 eV sufficed for well converged results. Due to the size of the unit cell, the reciprocal space was sampled at a single point (gamma). For the calculations of NO, spin-polarised calculations were required to account for the non-paired electron. NH_3 and bioNICS-1 have closed electronic shells. Calculations were performed using a Gaussian smearing of 0.03 eV.

Initially, a full geometric and structural optimization was performed, where the size of the unit cell and the atomic positions in bioNICS-1 were allowed to change, revealing the lattice parameters. The force threshold for optimization was set at 0.01 eV/Å. Having identified the optimum dimension of the unit cell, it was then kept fixed as several possible sites for NO and NH_3 adsorption were probed and two distinct (accounting for symmetry) were identified. The energy of adsorption was calculated as

$$\boldsymbol{E_{ads} = E_{bioNICS1+adsorbate} - E_{bioNICS1} - E_{gaseous}},$$

where $\boldsymbol{E_{bioNICS1+adsorbate}}$ is the electronic energy of bioNICS-1 with the adsorbate, $\boldsymbol{E_{bioNICS1}}$ is the energy of empty bioNICS-1 and $\boldsymbol{E_{gaseous}}$ is the energy of the unperturbed adsorbate molecule in vacuum. In all instances, a Grimme D3 correction to describe the van der Waals interactions were applied since vanilla DFT severely underestimates them.

To account for the temperature effects, the Gibbs free energies of the adsorption were also calculated. For bioNICS-1, we can assume that $\boldsymbol{G = E}$, as we have $\Delta\boldsymbol{S} \approx \boldsymbol{0}$ and $\Delta\boldsymbol{V} \approx \boldsymbol{0}$ upon adsorption. For the adsorbates, an ideal gas approximation is used in the gaseous phase (vibrationa, rotational, translational degrees of freedom) and the harmonic approximation is used in the adsorbed state, including the vibrational degrees of freedom only

To study the geometric parameters of the structure, a Monte Carlo simulation with 200.000 hits was performed on the DFT-optimized structure. Using Zeo++, which uses the Voronoi decomposition, we have calculated the pore diameter, surface area, accessible volume, pore-size distribution, stochastic ray tracing and probe-occupiable volume for bioNICS-1. The accessible surface area was evaluated for probes of various radii (0.5-3.5 Å).

<u>Time-resolved DRIFT spectroscopy</u> was performed on a Perkin Elmer model Frontier spectrometer equipped with a DiffusIR reaction chamber from Pike Scientific. Spectra were recorded using Timebase software and a liquid nitrogen cooled MCT detector. The analysed spectral range was between 1000 and 4000 cm^{-1}, spectral resolution of 4 cm^{-1} and 4 accumulations per scan, allowing us to achieve a time resolution of about 15 seconds. For the analysis, about 10 mg of powdered sample was positioned inside the ceramic sample holder and sealed with a KBr window. The samples were degassed overnight in vacuum (10^{-5} mbar, pump model Hi cube by Pfeiffer vacuum) at 120 °C. After degassing, the cell was pressurized with nitrogen (purity 5.0) to ambient pressure, and flow was maintained at 10 ml/min using Brooks electronic mass flow controllers (model 5850). After cooling the sample to 25 °C in N_2 flow, the background was recorded with a degassed sample, and spectral recording was initiated. After 15 minutes in N_2 flow, the sample was saturated with NO gas (1 ml/min, mixed with 10 ml/min N_2 flow) at 25°C for about 30 minutes. After NO saturation, the sample was

flushed in N_2 flow until removing all gas phase NO, and the NO desorption was initiated by moist N_2. Nitrogen was saturated with water vapor at 23 °C by passing the flow through a saturator.

Nitric Oxide (NO) release studies in the liquid phase were conducted using the oxyhaemoglobin assay as described by Feelisch et al.[41], which is highly specific for the detection and quantification of NO under aerobic conditions. This method is based on the basic reaction of oxyhaemoglobin (oxyHb) with NO, forming methaemoglobin (metHb) and nitrate.

oxyHb + NO → metHb + NO^{3-}

Polytetrafluoroethylene (PTFE) was added to activated bioNICS-1 samples at a ratio of 75% (sample): 25% (PTFE) (w/w). The mixture was compressed into discs (under 8 tons for 30 s) to avoid sample dispersion in the liquid phase. Discs weighing approximately 5 mg were inserted into a cell with a vacuum valve, followed by sample degassing for 2 h at 150 °C, with the vacuum greater than 10^{-2}Pa. After degassing, NO was introduced into the cell under a pressure of 80 kPa, the condition was maintained for three days by closing the valve and removing the cell from the vacuum line. After loading NO, the remaining NO in the cell was evacuated, and was immediately filled with helium to atmospheric pressure. The cell remained closed until the start of the oxyhaemoglobin test. Oxyhaemoglobin solution (oxyHb) was prepared using the previously described method.[42] Briefly, 20 mg of lyophilized human haemoglobin was dissolved in 1 mL of buffer solution, and sodium dithionite was added to ensure complete reduction of haemoglobin. Sephadex G-25 column was used for purification and removal of salts from the obtained oxyHb solution.

The NO release experiment was conducted at room temperature using a UV/Vis spectrophotometer (Genesys 10S, Thermo Scientific) and quartz cuvettes with a volume of 3 mL. The first spectrum measurement was performed with an oxyHb solution (1 µM) without the sample, serving as a reference. Subsequently, the cell containing the NO-loaded sample was opened, and the sample was immediately added to the cuvette with the oxyHb solution. Spectra were recorded every 15 min for 3 h. The kinetic profile of NO release was calculated according to the protocol presented in the seminal work of Feelisch et al.[41]

Nitric Oxide (NO) release studies in the liquid phase were also conducted using Griess assay.

NO loading in BioNICS (powder):

For the Griess assay, 5 mg of the BioNICS powder sample was measured in a glass cell which was then introduced into the adsorption line and put under high vacuum via a high vacuum pump system

composed of a turbomolecular pump and a diaphragm pump (Pfeiffer Vacuum). The sample was then outgassed for two hours at 150 ºC, after which NO was introduced to the system until a pressure of 80 kPa was achieved and left to adsorb for 3 days. After the NO loading period, the remaining NO inside the cell was evacuated under vacuum and immediately filled with helium up to atmospheric pressure to avoid premature release.

NO release:

The NO released over time from the material was quantified indirectly through its stable decomposition product, nitrite (NO_2^-), which accumulated in the liquid medium and was measured using the Griess method. The loaded material was incubated in a phosphate buffer (pH = 7.4) at 450 µg/mL. Samples were taken from the liquid medium after 30 min, 1 h, and then every hour until the sixth hour. The samples were then incubated with the Griess reagent (0.1 % naphthylethylenediamine dihydrochloride and 1 % sulphanilamide in 5 % phosphoric acid), producing a chromophoric azo product through the reaction with nitrite. The absorbance of this species was measured at 548 nm using a microplate reader (Tecan, A-5082 Sunrise Remote), and the values were converted into equivalent nitrite concentrations using a calibration curve prepared with sodium nitrite solution and the Griess reagent, following the same procedure used for the samples. The calibration curve is presented in Figure S27.

In vitro cytotoxicity

Cell Culture:

HaCaT cells, a spontaneously immortalized keratinocyte cell line derived from adult human skin (CLS 300493; CLS Cell Lines Service GmbH), were cultured in DMEM medium (Dulbecco's Modified Eagle Medium; Sigma) with 10% FBS (Fetal bovine serum; Sigma) under standard conditions (37 °C, 5 % CO_2). Cells were subcultured using trypsin (Sigma) before reaching confluence. Cells were used within 10 consecutive passages.

Stability of bioNICS1 in cell medium (in culture medium):

The stability of the activated material - bioNICS1 in the cell medium (culture medium) was tested under incubation conditions (37°C, 5% CO_2) for 24 and 48 h. We tested the highest concentration used - 900 µg/mL. The concentration of released Zn^{2+} was determined using the ICP-OES method.

Cytotoxicity Test:

Particle toxicity was assessed by monitoring changes in the metabolic activity of HaCaT cells 24 and 48 h after adding the MOF particle suspension. The day before the experiment, cells were seeded onto a white 96-well microplate (Costar) at a density of 20,000 cells per well in 100 µL of medium. The next

day, cells were washed twice with DMEM medium without supplements and then treated with the test suspension of MOF microparticles in a concentration range of 3.5-900 μg/mL (material suspended in colourless DMEM medium) and incubated for 24 or 48 h. After incubation, the metabolic activity of cells was determined using the PrestoBlueTM Cell Viability Reagent (Thermo Fischer Scientific), according to the manufacturer's instructions. Substrate conversion was determined by fluorescence measurement. The relative metabolic activity of exposed cells to particles was calculated as the percentage of metabolic activity of non-exposed control cells.

4. Structural examination

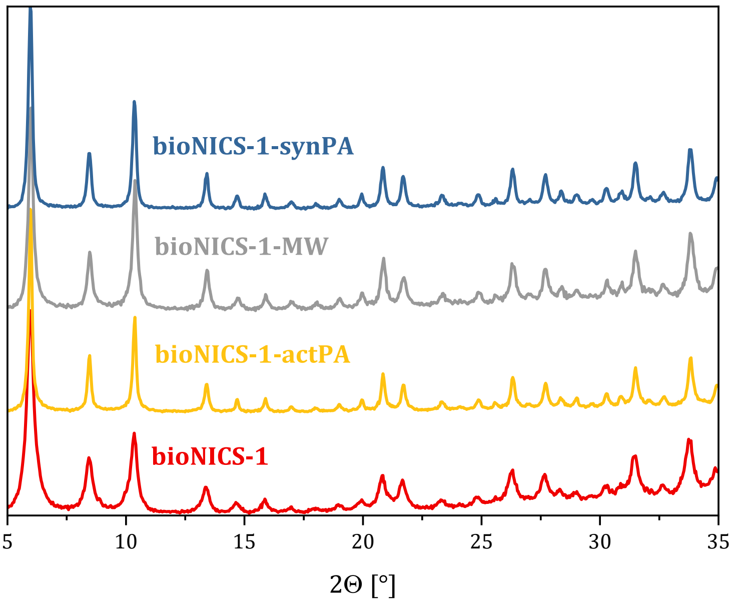

Figure S1: PXRD diffractograms of materials prepared according to protocols described above.

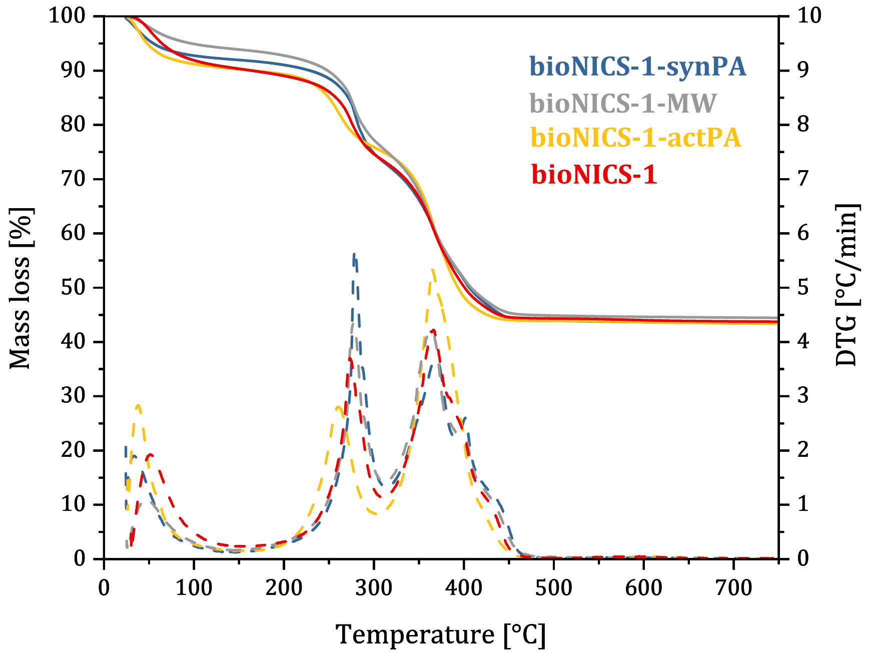

Figure S2: TGA/DTG of materials prepared according to protocols described above.

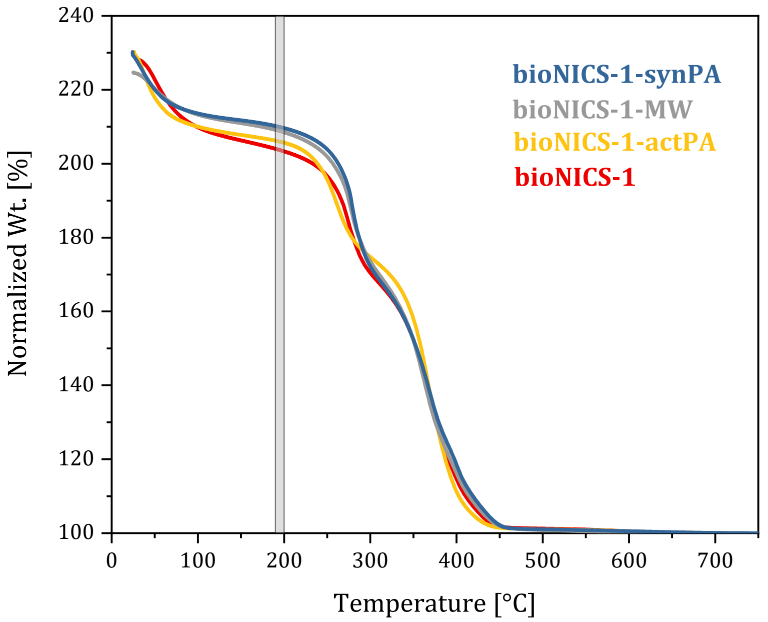

Figure S3: Normalized weight loss; ZnO residue representing 100%. The gray strip represents the temperature range where ascorbic acid begins to decompose[3] and marks the start of bioNICS-1 degradation.

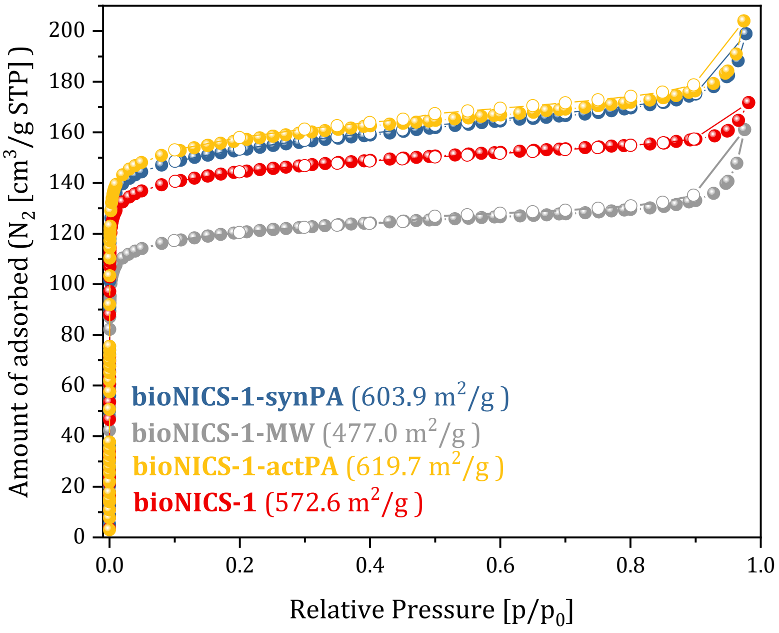

Figure S4: N_2 isotherms with measured BET specific surface area of corresponding sample.

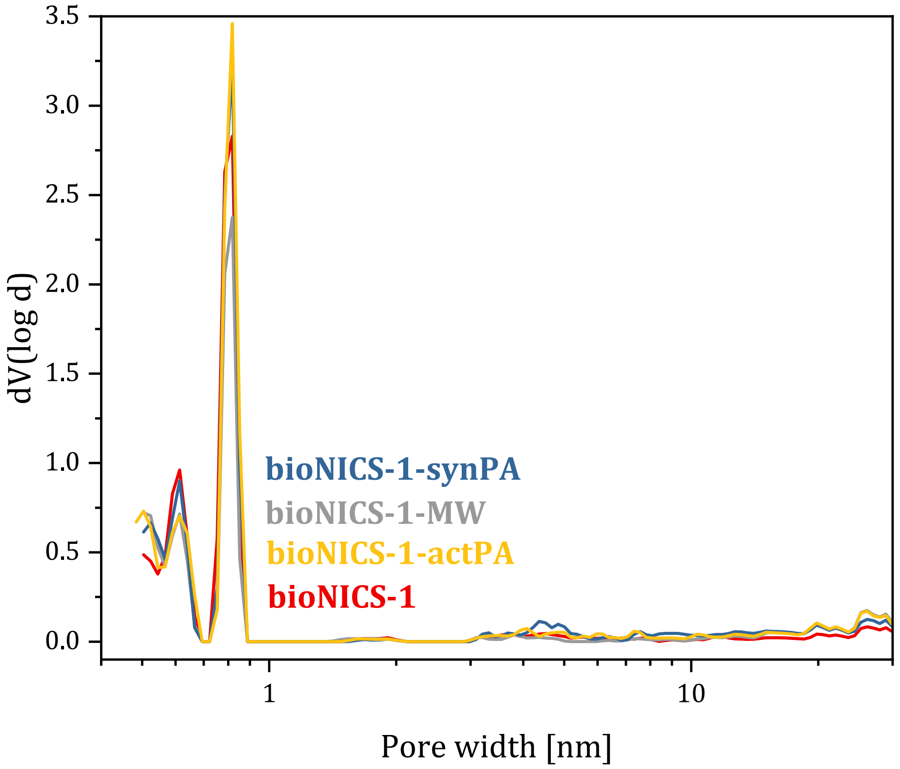

Figure S5: Pore size distribution of materials prepared according to protocols described above.

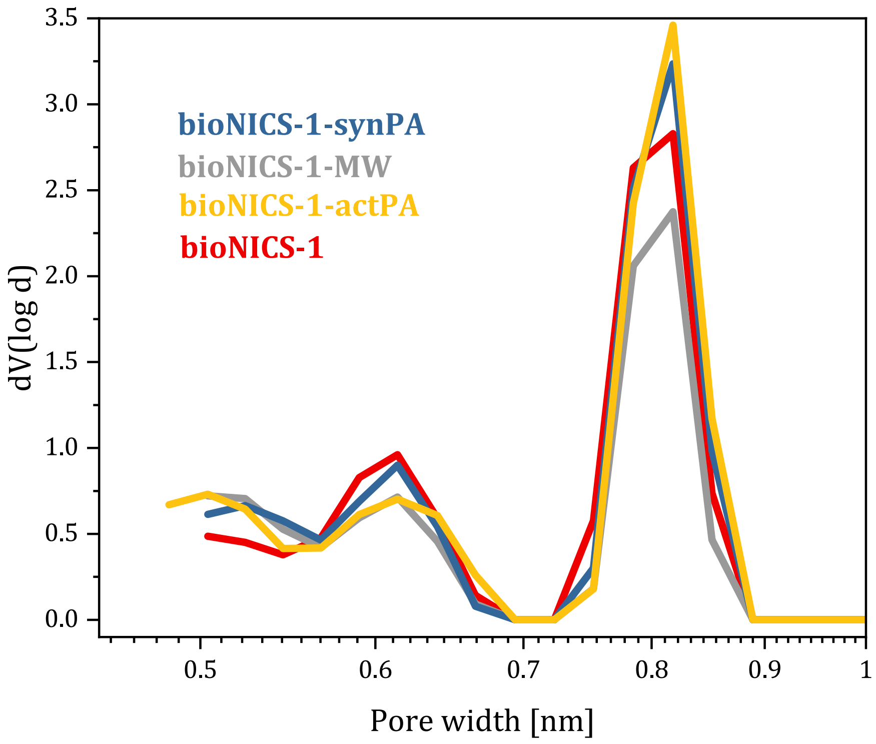

Figure S6: Close up of smaller voids in the sample, and possible additional ultra micro pore in bioNICS-1-actPA sample.

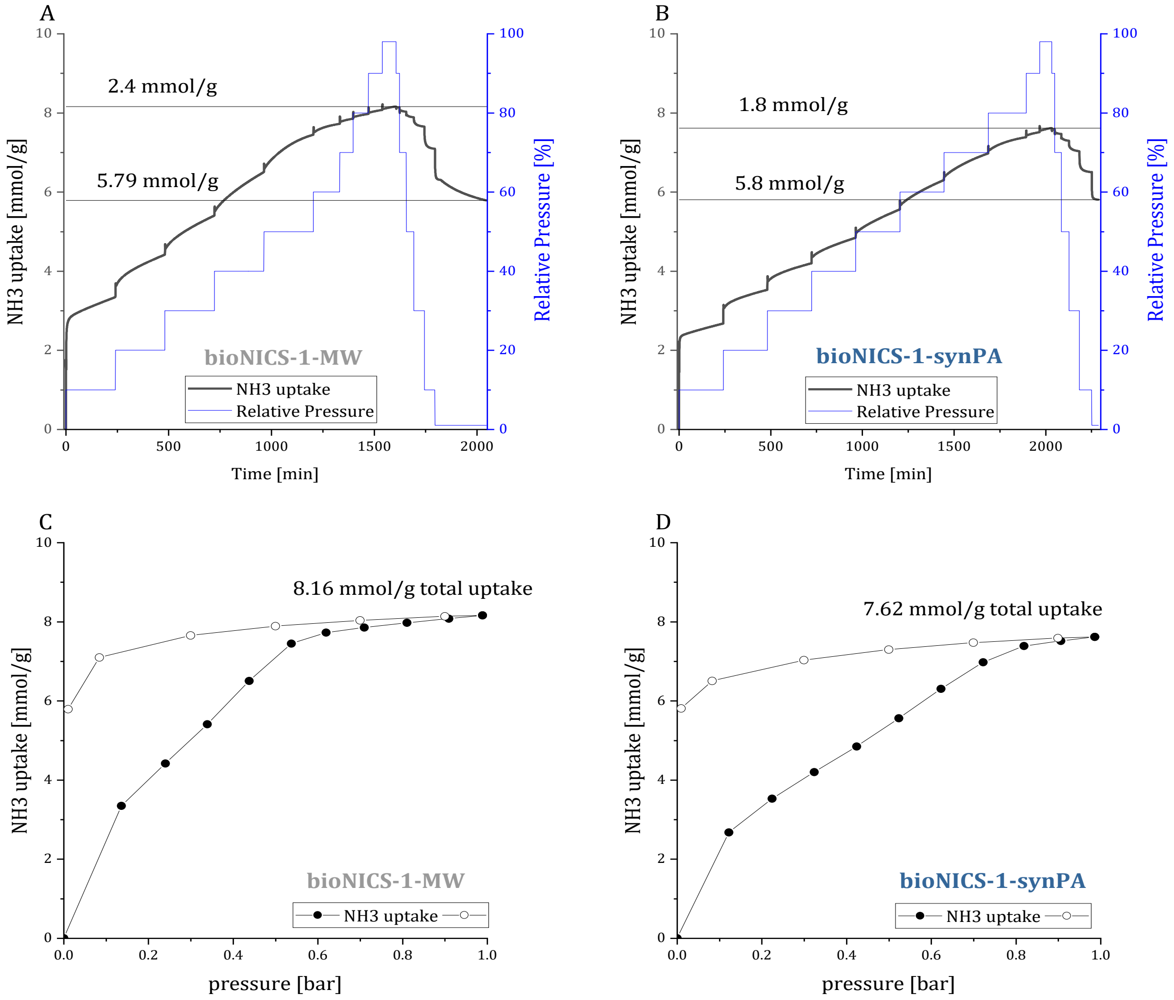

Figure S7: Ammonia sorption kinetics profile of selected materials (A and C) bioNICS1-MW, (B and D) bioNICS1-synPA. The difference between total uptake and the amount retained after the desorption step represents weakly bound or physiosorbed ammonia. The residual amount is so attributed to chemisorbed ammonia which also represents the amount of available acid sites in the structure.

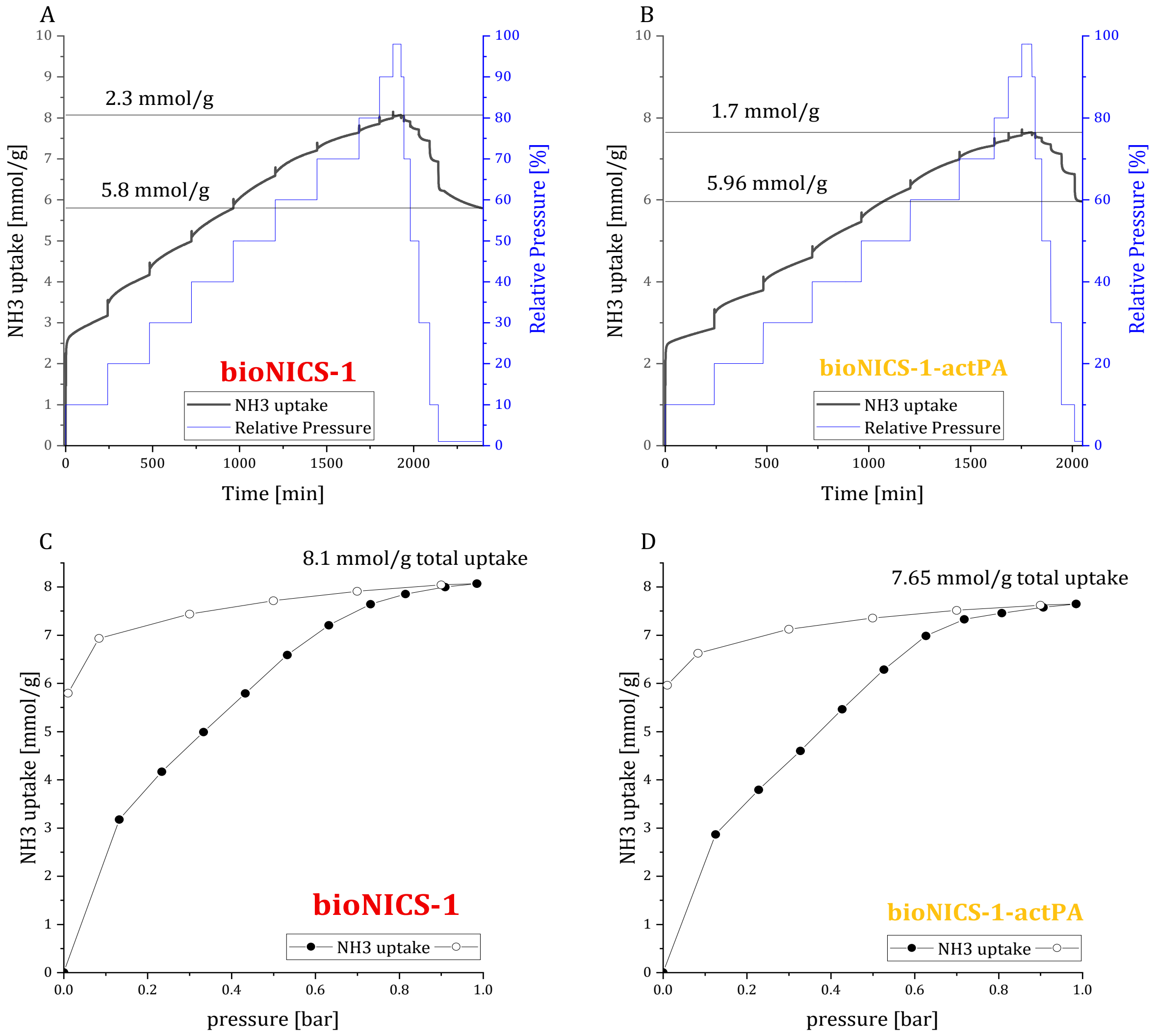

Figure S8: Ammonia sorption kinetics profile of selected materials (A and C) bioNICS1-1, (B and D) bioNICS1-actPA. The difference between total uptake and the amount retained after the desorption step represents weakly bound or physiosorbed ammonia. The residual amount is so attributed to chemisorbed ammonia which also represents the amount of available acid sites in the structure.

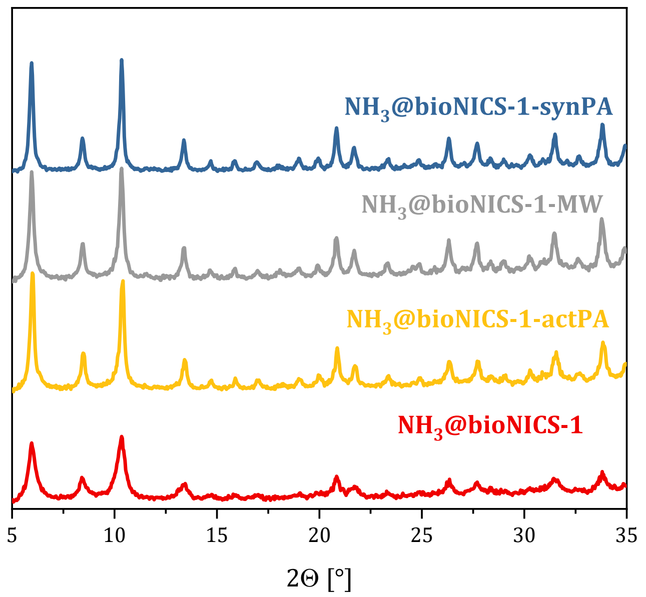

Figure S9: PXRD diffractograms of materials **after NH_3 sorption** experiment showing no changes in the structure.

5. NO sorption mechanism

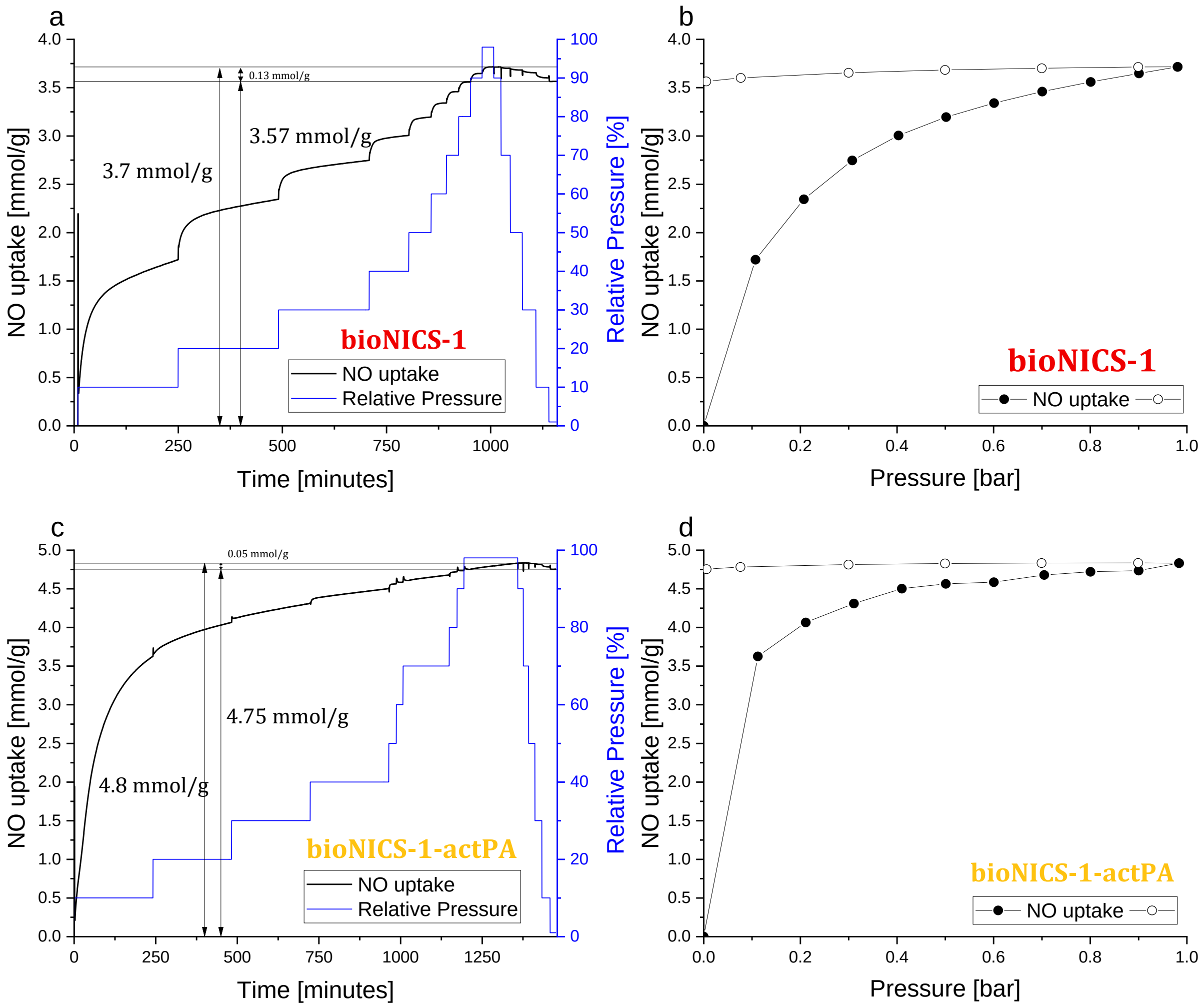

Figure S10: Nitric oxide sorption kinetics profile of (a and b) bioNICS-1, and (c and d) bioNICS-1-actPA. The difference between total uptake and the amount retained after the desorption step represents weakly bound or physiosorbed NO, the amount of which is approximately the same for all the samples. The residual amount is so attributed to chemisorbed NO

Analysis of sorption kinetics - Linear Driving Force (LDF) model and Avrami model.

$$Q = Q_{eq}(1 - e^{-k.t}) \qquad \text{Eq1}$$

$$Q = Q_{eq}(1 - e^{-(k.t)^n}) \qquad \text{Eq2}$$

Q represents adsorbed amount (mmol/g) at given time *t* (min). Q_{eq} is the maximum equilibrium adsorption capacity at defined conditions, *k* is overall mass transfer coefficient (min^{-1}) governed by pored diffusion, particle size and gas-solid interactions, *n* is Avrami exponent, describing the mechanism of adsorption.

Table S3: Fitting parameters for linear driving force (Eq1) and Avrami (Eq2) models.

Fitting model	Parameters	**Sample**	
		**bioNICS-1**	**bioNICS-1-actPA**
LDF	Q_{eq} (mmol/g)	1.592 ± 0.004	3.096 ± 0.003
	k (min^{-1})	0.0373 ± 0.0005	0.01451 ± 0.00004
	R^2	0.9212	0.9984
Avrami	Q_{eq} (mmol/g)	1.751 ± 0.005	3.108 ± 0.006
	k (min^{-1})	0.136 ± 0.002	0.0150 ± 0.0002
	n	0.573 ± 0.005	0.990 ± 0.004
	R^2	0.9928	0.9984

6. High-angle annular dark field scanning transmission electron microscopy

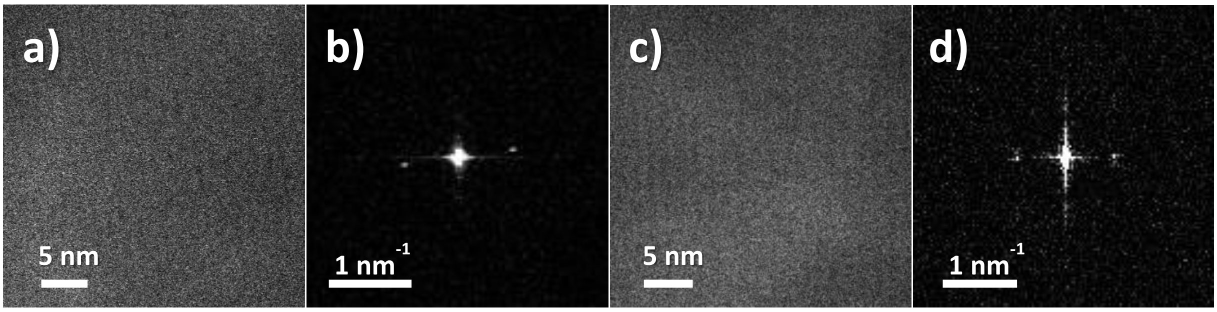

Figure S11: HAADF-STEM images of pristine bioNICS-1 before (a) and after (c) NO treatment. The corresponding fast Fourier transforms (FFTs) of images (a) and (c) are shown in (b), and (d), respectively. The HAADF-STEM images in (a) and (c) were acquired simultaneously with high-pass filtered iDPC images from the same regions.

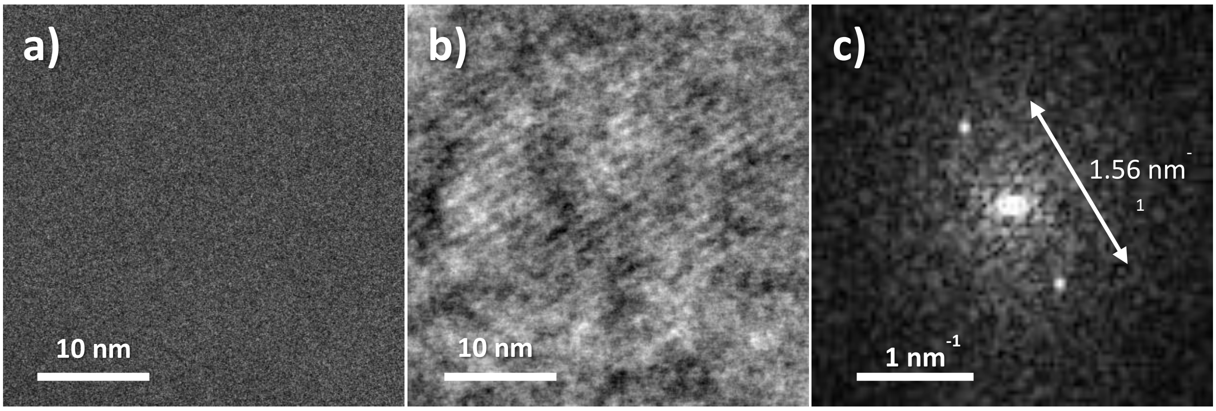

Figure S12: An additional HAADF-STEM (a) and a high-pass filtered (sigma of 80 pixels) iDPC image (b) of bioNICS-1 after NO treatment, acquired simultaneously from the same region. The corresponding FFT of image (b) is shown in (c), where the measured distance of 1.56 nm^{-1} between the reflections (c) corresponds to a d-spacing of 1.28 nm.

7. FTIR/DRIFTS *in situ* measurements

Fresh samples (Figure S13) are characterized by a broad absorption band between 2700 and 3800 cm^{-1}, characteristic of hydrogen-bonded OH groups in organic acids. Additionally, bands for C-H stretching at 2869 cm^{-1}, intense C=O and C=C ring vibrations at 1715 and 1653 cm^{-1}, CH_2 wagging at 1402 cm^{-1}, C-H bending at 1344 cm^{-1} and C-O-C stretching at 1101 cm^{-1} are visible, which is in accordance with characteristic IR spectrum of ascorbic acid.[4] The largest spectral change in the samples after the NO chemisorption/desorption is the attenuation of the C-H vibrations between 2850-2950 cm^{-1} and the blue shift of the C=O band vibration from 1715 to 1764 cm^{-1}. This reveals chemical changes of the bioNICS-1 sample, very likely related to oxidation of the C-H and O-H functionalities.

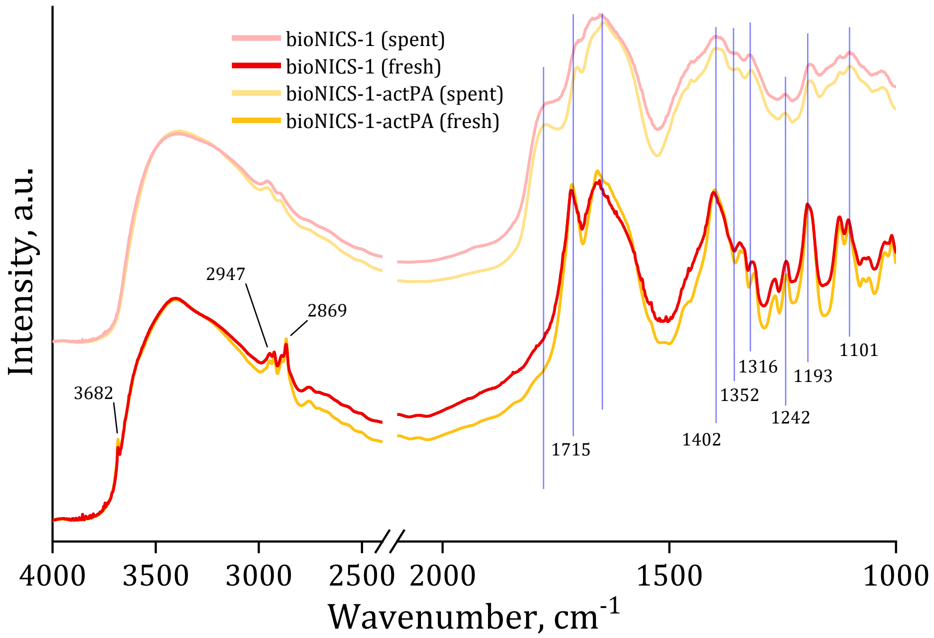

Figure S13: DRIFT spectra for bioNICS-1, and bioNICS-1-actPA samples before and after NO chemisorption/desorption experiments. Spectra are offset vertically for clarity.

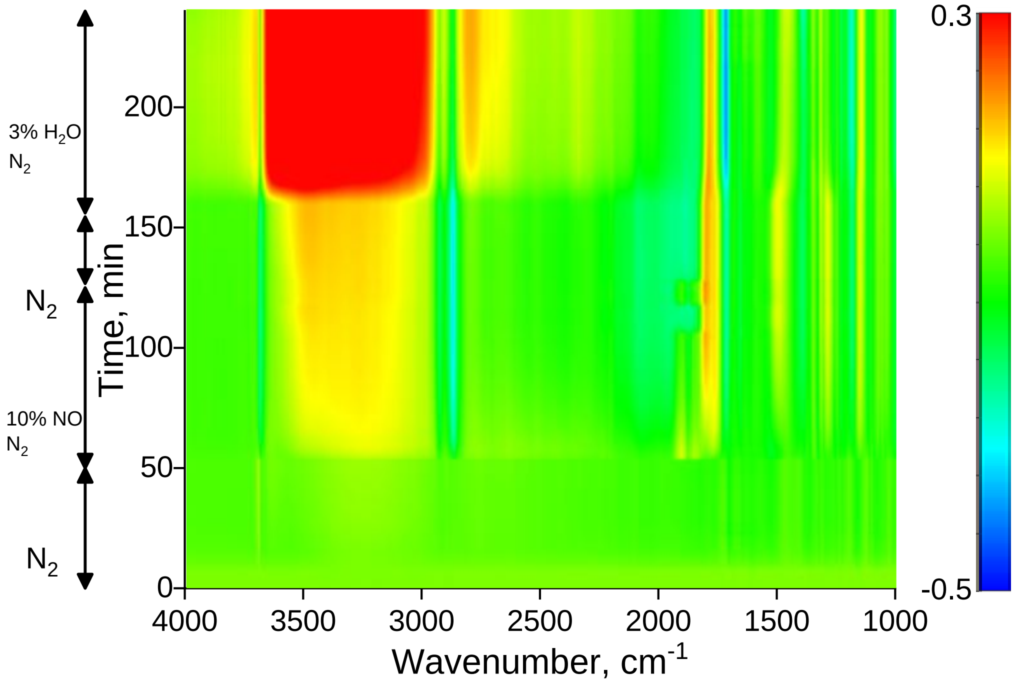

Figure S14: Time-resolved DRIFTS 2D map for the NO saturation/desorption from the **bioNICS-1** sample. Time-lapse of the experiment: Sample cooling from 120°C to RT and flushing with N_2 at room temperature from 0 min to 53 min. From 53 min to 118 min, the sample was saturated with 10% NO, degassing sample in dry N_2 from 118 min to 156 min, and sample degassing with wet N_2 from 156 to 240 min.

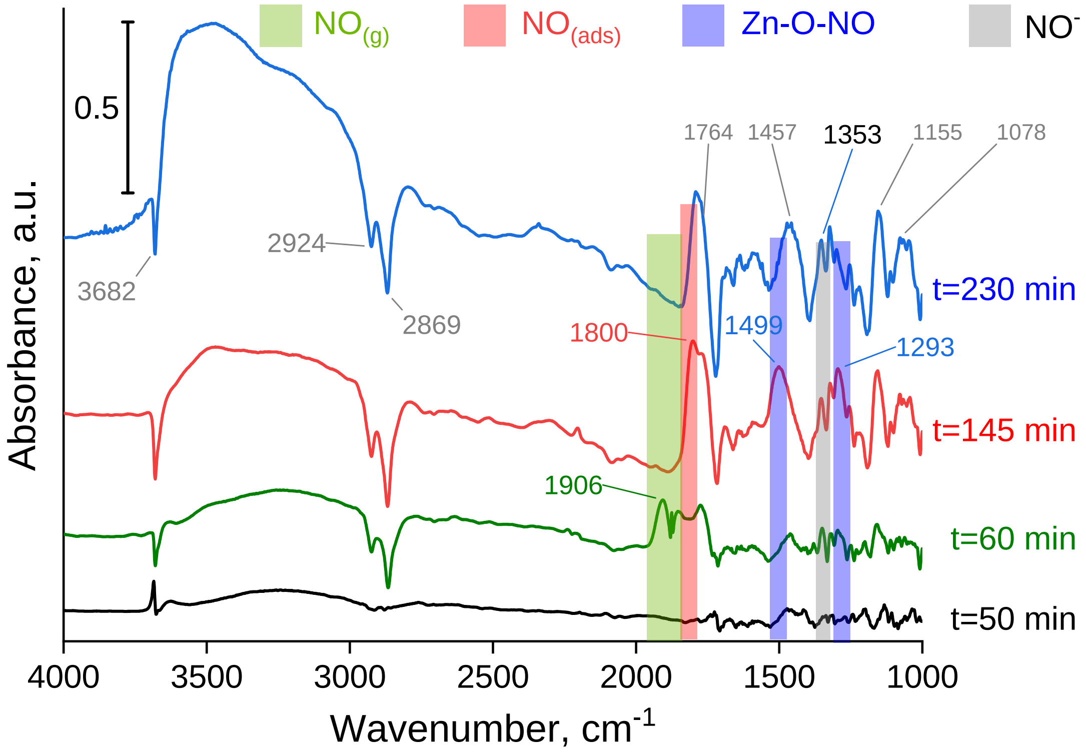

Figure S15: DRIFT spectra of the **bioNICS-1** sample after degassing in N_2 (50 min), during saturation with 10% NO (60 min), after desorption in N_2 (145 min), and after desorption in 3% H_2O/N_2 flow (230 min). Colour bars highlight the most intense vibrations of different NO species.

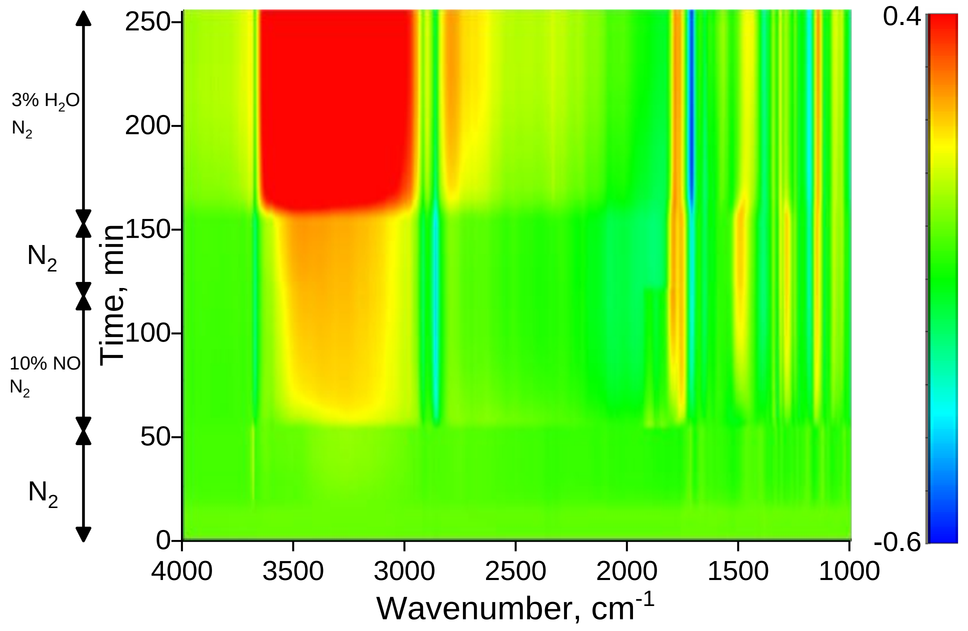

Figure S16: Time-resolved DRIFTS 2D map for the NO saturation/desorption from the **bioNICS-1-actPA** sample. Time-lapse of the experiment: Sample cooling from 120°C to RT and flushing with N_2 at room temperature from 0 min to 53 min. From 53 min to 118 min, the sample was saturated with 10% NO, degassing sample in dry N_2 from 118 min to 153 min, and sample degassing with wet N_2 from 153 to 256 min.

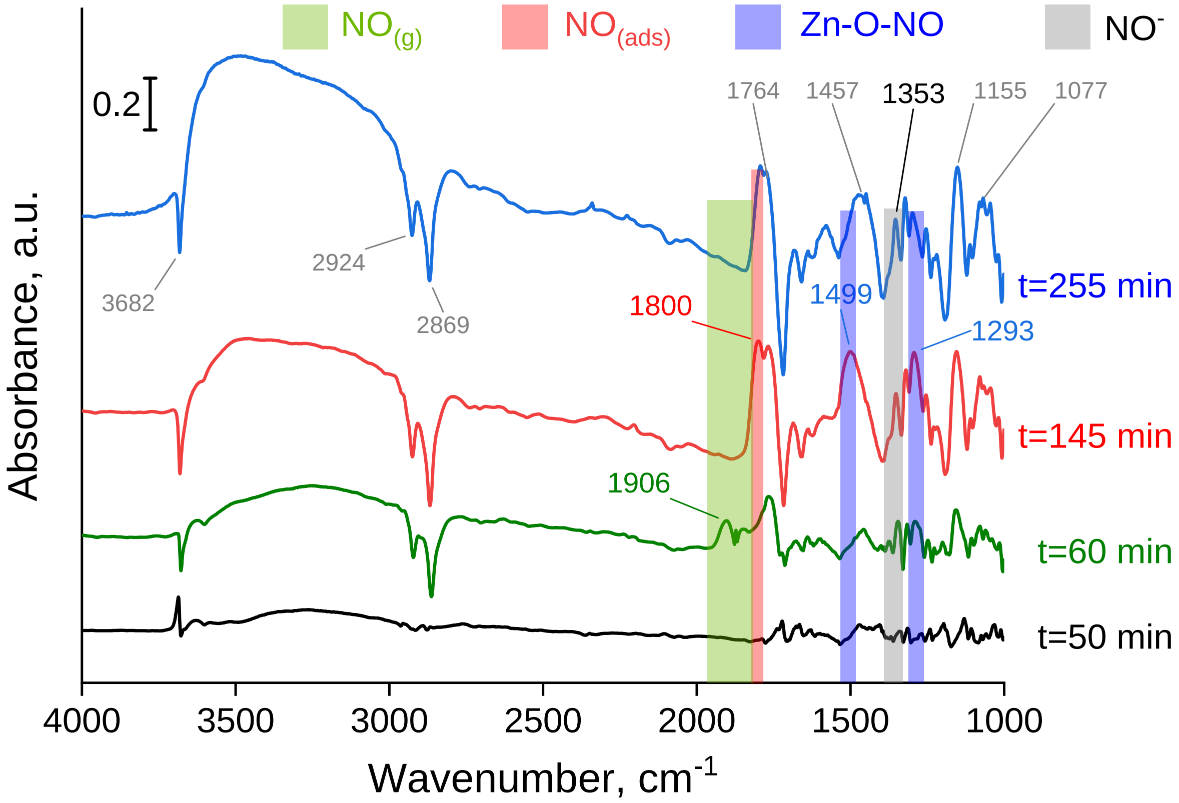

Figure S17: DRIFT spectra of the **bioNICS-1-actPA** sample after degassing in N_2 (50 min), during saturation with 10% NO (60 min), after NO desorption in dry N_2 (145 min), and after NO desorption in 3% H_2O/N_2 flow (255 min). Colour bars highlight the most intense vibrations of different NO species.

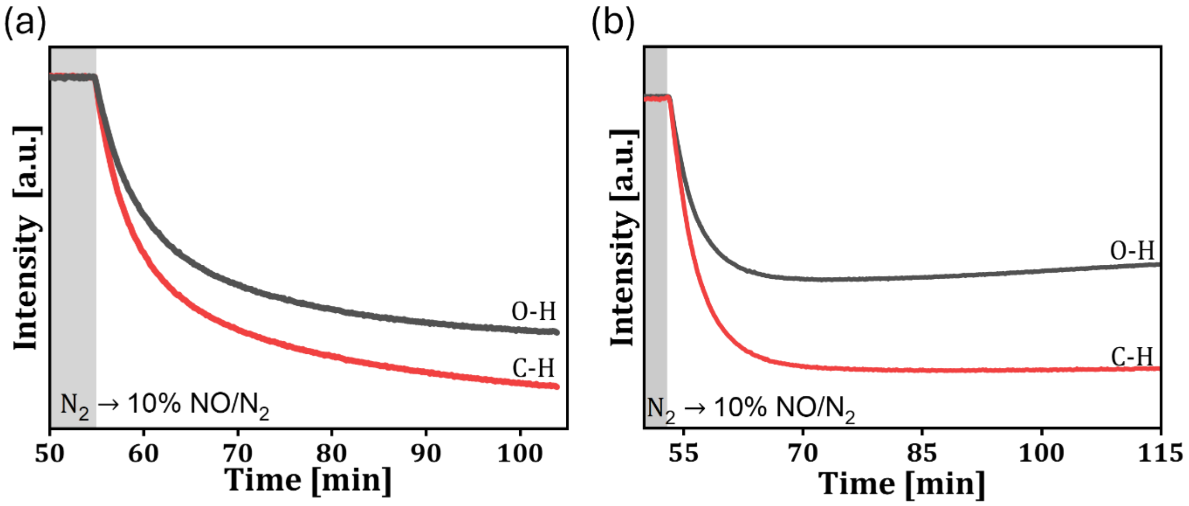

Figure S18: Time-resolved DRIFT profiles during sample saturation with NO (N_2 → 10 %NO/N_2 step change) showing the decrease of C-H (2869 cm^{-1}) and O-H (3682 cm^{-1}) band intensity – (a) bioNICS-1 and (b) bioNICS-1-actPA.

Computational method

To better understand the geometry of bioNICS-1, DFT optimisation was performed on the experimentally determined structure, as observed by X-ray. A cubic unit cell with a dimension of 21.11 Å was found with a chemical formula $Zn_{44}C_{144}O_{152}H_{120}$, which yields a material density of 1.27 g cm^{-3}.

The surface area of channels was determined as 1625 m^2 cm^{-3} and the dimensions of the pore are 8.6 x 7.5 x 8.6 Å^3, yielding an accessible volume fraction of 0.111. The channels are accessible to a spherical probe of a maximum radius of 3.67 Å, which puts a limit on the adsorbate size. The accessible surface area is a function of the probe size, as shown in Figure S19. For a probe with a size of 2 Å, which corresponds roughly to the hydrodynamic radius of NO, the accessible surface area is 1250 m^2 g^{-1}.

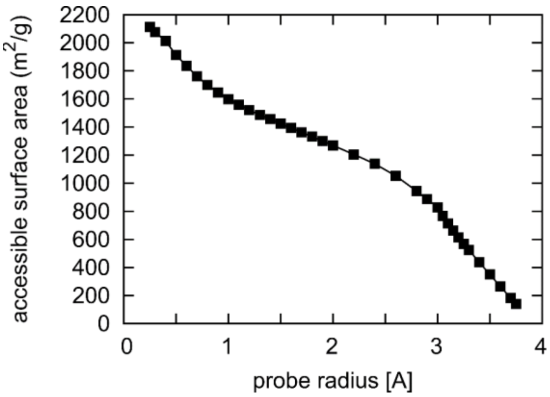

Figure S19: The accessible surface area of bioNICS-1 as a function of the probe radius, as calculated using Monte Carlo.

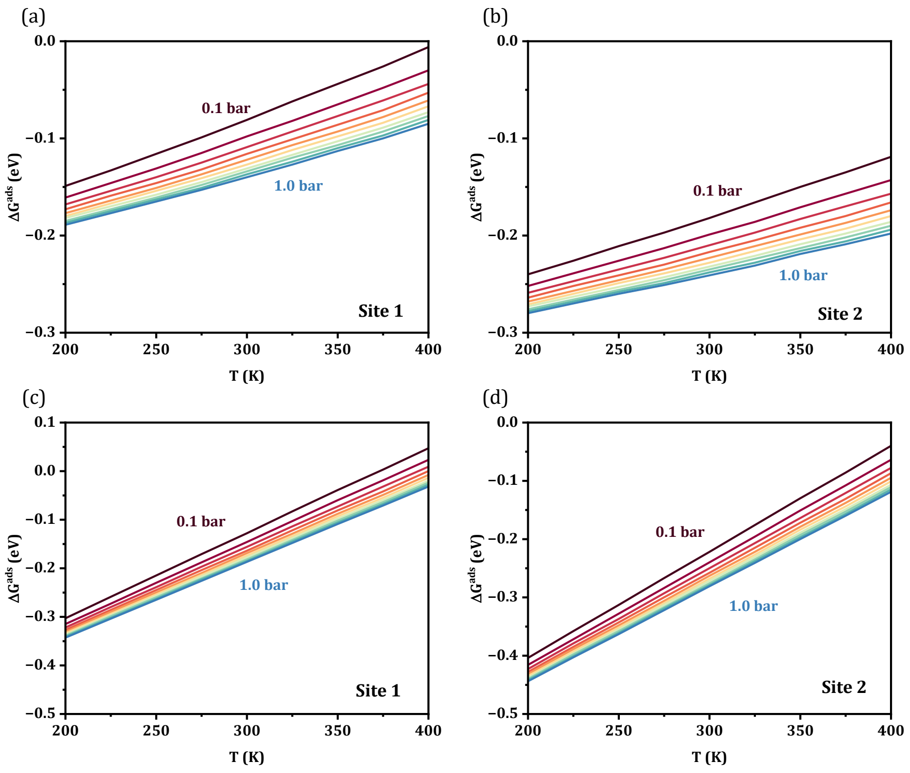

Figure S20: Gibbs free energies of adsorption of NO and H_2O on sites 1 (a) and (c), and sites 2 (b) and (d), as a function of temperature and pressure (step of 0.1 bar).

8. Oxyhaemoglobin and Griess assay

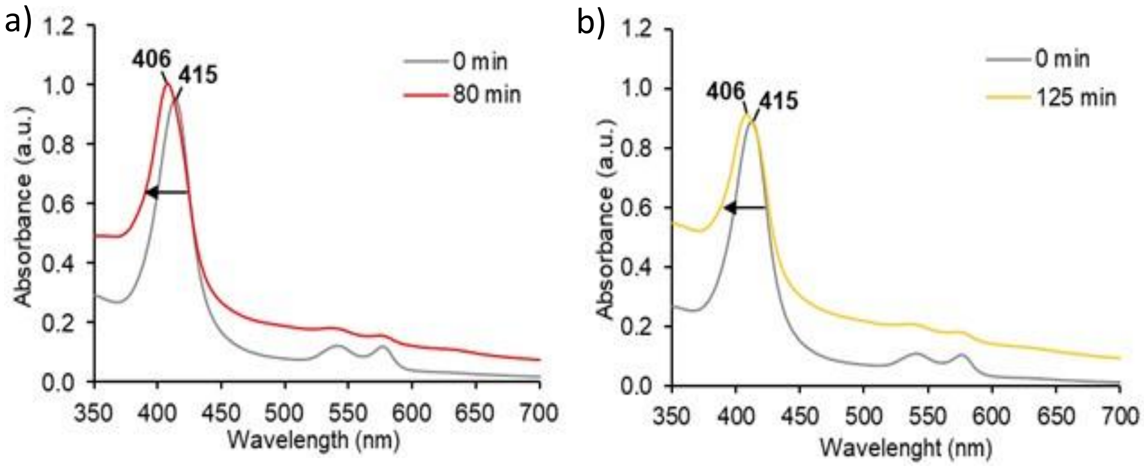

Figure S21: Evolution of the UV-VIS spectrum over time in the oxyhaemoglobin assay: Comparison between the initial UV-VIS spectrum of the oxyhaemoglobin solution and the final stabilized spectrum with NO-loaded (a) bioNICS-1 and (b) bioNICS-1-actPA.

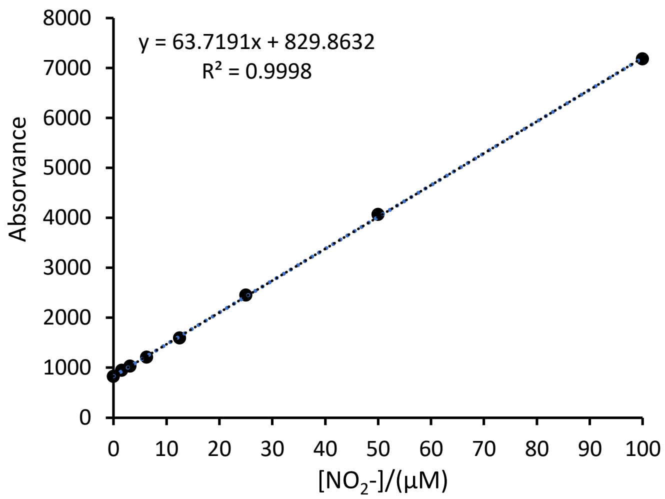

Figure S22: Calibration curve obtained using a $NaNO_2$ solution.

9. Preliminary *in vitro* study

Despite the material exhibiting satisfactory stability in aqueous environments, it is acknowledged that the cell culture medium is a highly complex milieu, and with an increase in the ionic strength of the environment, the dissolution of the material occurs more rapidly. To assess the stability of unmodified bioNICS-1 in the culture medium, a concentration of 900 µg/mL was utilized, with Zn^{2+} constituting 0.402 fraction (or 40%) of the framework. After 24h and 48h exposure of the material to the culture medium (without cells) under standard conditions, it is evident that 45-50% of Zn^{2+} is released from the bioNICS-1 framework during the experiment, or 41.2% for larger particles (Table S4).

Table S4: Concentrations of eluded zinc [ppm] after 24 or 48h of exposure to cell culture medium

Sample	Zn^{2+} [ppm]
bioNICS-1+ cell culture medium 24h	166
bioNICS-1+ cell culture medium 48h	183
bioNICS-1+ cell culture medium 48h – larger particles	149

In accordance with literature sources[5], the toxicity of Zn^{2+} (expressed as the IC50 value) is approximately 1.5 x 10^{-4} mol/L (or 9.807 µg/mL). Considering the percentage of released Zn^{2+} after 24 or 48 hours, the theoretical IC50 when using bioNICS-1 would be reached at a concentration of 48.21–53.15 µg/mL, or 59.21 µg/mL for larger particles. Experimental results align with theoretical predictions in experiments with smaller particles after 24 hours. However, when using larger particles (after 24 hours), the IC50 is achieved at a higher concentration than theoretically predicted (Figure S23a and b). We infer that particle size influences the dissolution kinetics and, consequently, the rate of Zn^{2+} release.

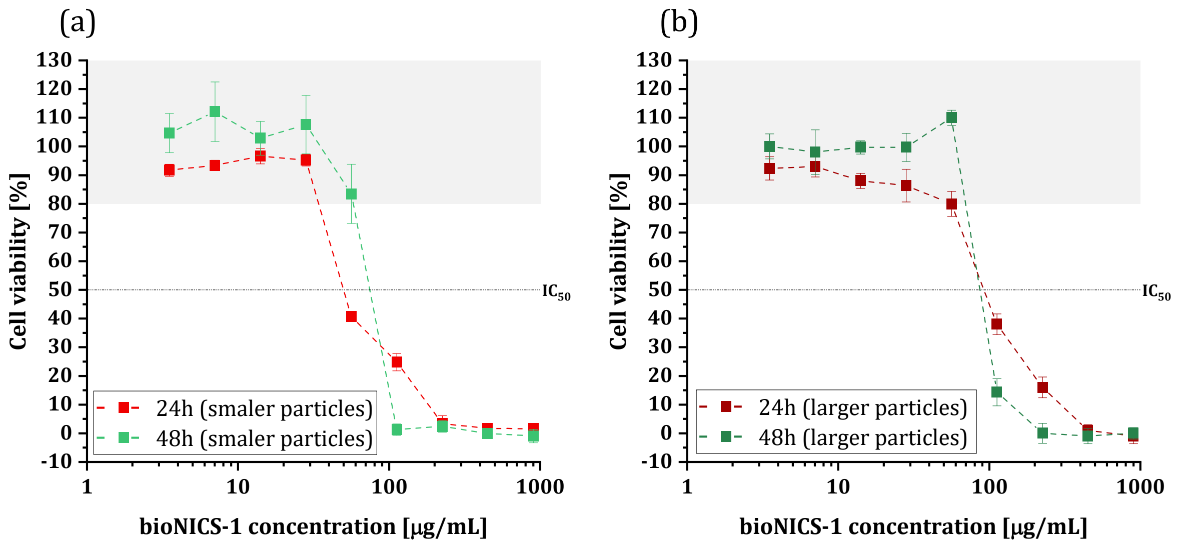

Figure S23: *in vitro* cytotoxicity test of smaller and larger bioNICS-1 particles on HaCAT cell line.

According to ISO10993-5 standard, cell viability above 80% represents non-toxic concentrations. Viability between 80-60% indicates weak toxicity, 60-40% moderate toxicity, and below 40% strong toxicity for the tested material concentrations[6]. The results indicate that the safe (tested)

concentration of the material for use on the HaCat cell line is 28.1 µg/mL (smaller particles) and 56.3 µg/mL (larger particles). Comparing the expected and determined IC50 values, we conclude that the toxic effect of bioNICS-1 is due to released Zn^{2+} ions.

Although bioNICS-1 may exhibit toxicity to the HaCat cell line at concentrations higher than (approximately) 30 µg/mL, the obtained IC50 values suggest that, compared to other Zn-MOFs, bioNICS-1 can be classified as a more toxicologically safe bioMOF based on Zn^{2+}. Assuming that 30 µg/mL of bioNICS-1 is a safe concentration (for the HaCat cell line), this quantity of material could deliver 0.154 µmol NO / mL. With the available concentration of NO for release, we are on the border between tissue regeneration and antimicrobial action.

10. Literature

[1] T. K. Tajnšek, N. Zabukovec Logar, M. Mazaj, *Molecules* **2023**, *28*, 253.

[2] T. K. Tajnšek, E. Svensson Grape, T. Willhammar, T. Antonić Jelić, U. Javornik, G. Dražić, N. Zabukovec Logar, M. Mazaj, *Commun. Chem.* **2022**, *5*, 24.

[3] S. Jingyan, L. Yuwen, W. Zhiyong, W. Cunxin, *J. Pharm. Biomed. Anal.* **2013**, *77*, 116–119.

[4] V. Sreeja, K. N. Jayaprabha, P. A. Joy, *Appl. Nanosci.* **2015**, *5*, 435–441.

[5] J. Borovanský, P. A. Riley, *Chem. Biol. Interact.* **1989**, *69*, 279–291.

[6] J. López-García, M. Lehocký, P. Humpolíček, P. Sáha, **2014**, *5*, DOI 10.3390/JFB5020043.
